# Identification of two miRNAs regulating cardiomyocyte proliferation in an Antarctic icefish

**DOI:** 10.1016/j.isci.2024.110128

**Published:** 2024-05-27

**Authors:** Qianghua Xu, Ruonan Jia, Fei Yang, Peng Hu, Xue Li, Saiya Ge, Shouwen Jiang, Jiulin Chan, Wanying Zhai, Liangbiao Chen

**Affiliations:** 1 Key Laboratory of Sustainable Exploitation of Oceanic Fisheries Resources, Ministry of Education, College of Marine Sciences, Shanghai Ocean University, Shanghai, China; 2 International Research Center for Marine Biosciences (Ministry of Science and Technology), Shanghai Ocean University, Shanghai 201306, China; 3 Key Laboratory of Aquaculture Resources and Utilization, Ministry of Education, College of Fisheries and Life Sciences, Shanghai Ocean University, Shanghai, China

**Keywords:** Molecular biology, Evolutionary ecology, Cell biology

## Abstract

The hemoglobinless Antarctic icefish develop large hearts to compensate for reduced oxygen-carrying capacity, which serves as a naturally occurred model to explore the factors regulating cardiogenesis. Through miRNAome and microRNAome comparisons between an icefish (*Chionodraco hamatus*) and two red-blooded notothenioids, we discovered significant upregulation of factors in the BMP signaling pathways and altered expression of many miRNAs, including downregulation of 14 miRNAs in the icefish heart. Through knocking down of these miRNAs, we identified two of them, miR-458-3p and miR-144-5p, involved in enlarged heart development. The two miRNAs were found to regulate cardiomyocyte proliferation by targeting bone morphogenetic protein-2 (*bmp2*). We further validated that activation of the miRNA-*bmp2* signaling in the fish heart could be triggered by hypoxic exposure. Our study suggested that a few miRNAs play important roles in the hypoxia-induced cardiac remodeling of the icefish which shed new light on the mechanisms regulating cardiomyocyte proliferation in heart.

## Highlights

• The icefish’s enlarged heart is caused by increased cardiomyocyte proliferation
• MiR-458-3p and 144-5p are involved in cardiomyocyte proliferation by targeting to BMP2
• Cardiomyocyte proliferation could be induced by the hypoxic condition in icefish

## Graphical abstract

**Figure:**
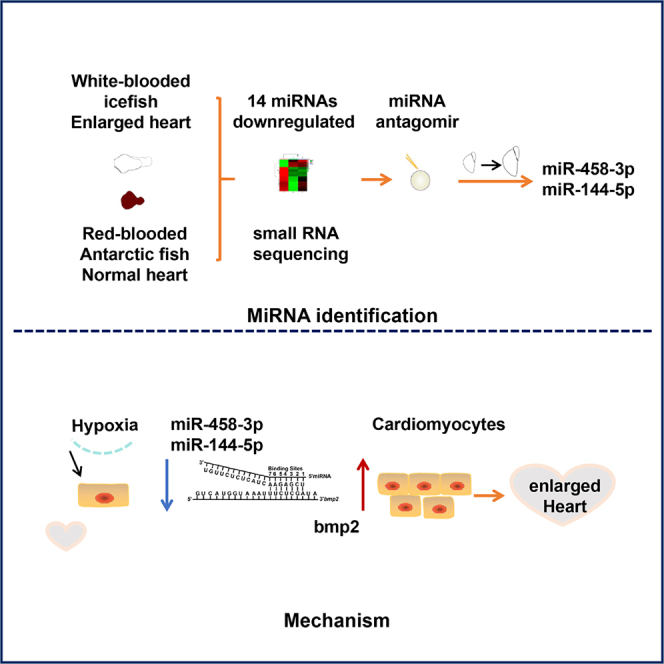

## Introduction

Fishes of the perciformes suborder Notothenioidei are the dominant fishes of the Southern Ocean, where they thrive because of a suite of adaptations to life at near-freezing temperatures.^1^^,^^2^ One of the most curious physiological traits of notothenioid fishes is found in the Family Channichthyidae, the white-blooded icefishes. Antarctic icefishes are the only known vertebrates to lack the circulating oxygen-binding protein hemoglobin (Hb) and functionally active erythrocytes.^3^^,^^4^ Fifteen of the 16 members of the family completely lack the adult β-globin gene and retain only a small 3′-fragment of the α-globin gene.^5^ Lack of Hb expression in icefish has been traced to a gene deletion event^6^^,^^7^ that occurred approximately 8.5 Ma when icefishes diverged from the red-blooded bathydraconids.^8^ In icefish, oxygen is carried throughout the circulatory system freely dissolved in the blood plasma; consequently, the oxygen-carrying capacity of icefish blood is only <10% of typical fish blood.^9^^,^^10^^,^^11^ To facilitate delivery of oxygen to the tissues, substantial remodeling of the heart and blood vessels of icefish has occurred.^12^^,^^13^ Also, many studies have suggested that the loss of Hb and Mb may have accelerated the development and evolution of these cardiovascular modifications.^14^^,^^15^^,^^16^ The enlarged heart, especially the ventricle, represents a key evolutionary adaptation for enhancing oxygen transport.^17^^,^^18^ The ventricle of icefish is three times larger than the ventricle of similar-sized red-blooded notothenioids.^2^ The ventricle myocardium of icefish is entirely trabecular and sponge-like, which is an advantageous feature good for limiting the harmful effects of dilated/hypertrophied type of cardiac adaptation when oxygen supply is limited.^18^^,^^19^ The icefish heart provides an excellent model system for investigating adaptive remodeling of heart developmental programs. However, the molecular pathways involved in the cardiogenetic processes in icefish heart are yet to be revealed.

Heart development in vertebrates is a complex morphogenetic process involving tightly controlled migration, specification, and proliferation of various cardiogenic cells.^20^ Among the cardiogenic cells, the cardiomyocytes originating from the myocardial progenitor cells constitute the majority of the cell population in the developed heart and are largely responsible for determining heart size.^21^ At the earliest developmental stages in zebrafish, the mode of cardiac growth is through addition of differentiating cells from outside the developing heart.^22^^,^^23^ Forty-eight hours postfertilization (hpf), cardiac growth then switches to proliferation of existing cardiomyocytes.^24^ Unlike mammals, in which cardiomyocytes proliferate only in early development and for a short period after birth, many fishes retain a cardiomyocyte proliferative capability into their adult life.^25^ Such a property makes it possible to investigate the regulatory programs of cardiomyocyte proliferation using adult fish. This is especially advantageous for the Antarctic icefish, for which developing embryos are rarely available for study.

To elucidate the signaling pathways responsible for the adaptive reprogramming of heart development in the icefish that leads to cardiac enlargement, we first examined the morphological appearance of the cardiac cells, then conducted mRNAome and microRNAome comparisons between the hearts of an icefish (*Chionodraco hamatus*) and two red-blooded notothenioids (*Trematomus bernacchii* and *Gymnodraco acuticeps*) followed with *in vitro* and *in vivo* functional studies of differentially expressed miRNAs and target genes. The white-blooded *C*. *hamatus* and the two red-blooded *T*. *bernacchii* and *G*. *acuticeps* belong to the Perciformes suborder Notothenioidei, and the genetic evolutionary tree for the three Antarctic fish species is shown on Figure S1. We revealed that hypoxia-induced downregulation of two miRNAs, miR-458-3p and miR-144-5p, is linked to elevated cardiomyocyte proliferation and enlarged heart, through relaxed suppression of *bmp2* signaling. Our discovery of a molecular cascade that fosters enlargement of the icefish heart may provide insights into potential mechanisms of cardiac regeneration in humans and other vertebrates.

## Results

### Morphological comparison between the white-blooded *C. hamatus’* heart and red-blooded notothenioid *T. bernacchii’s* heart

**Figure 1:**
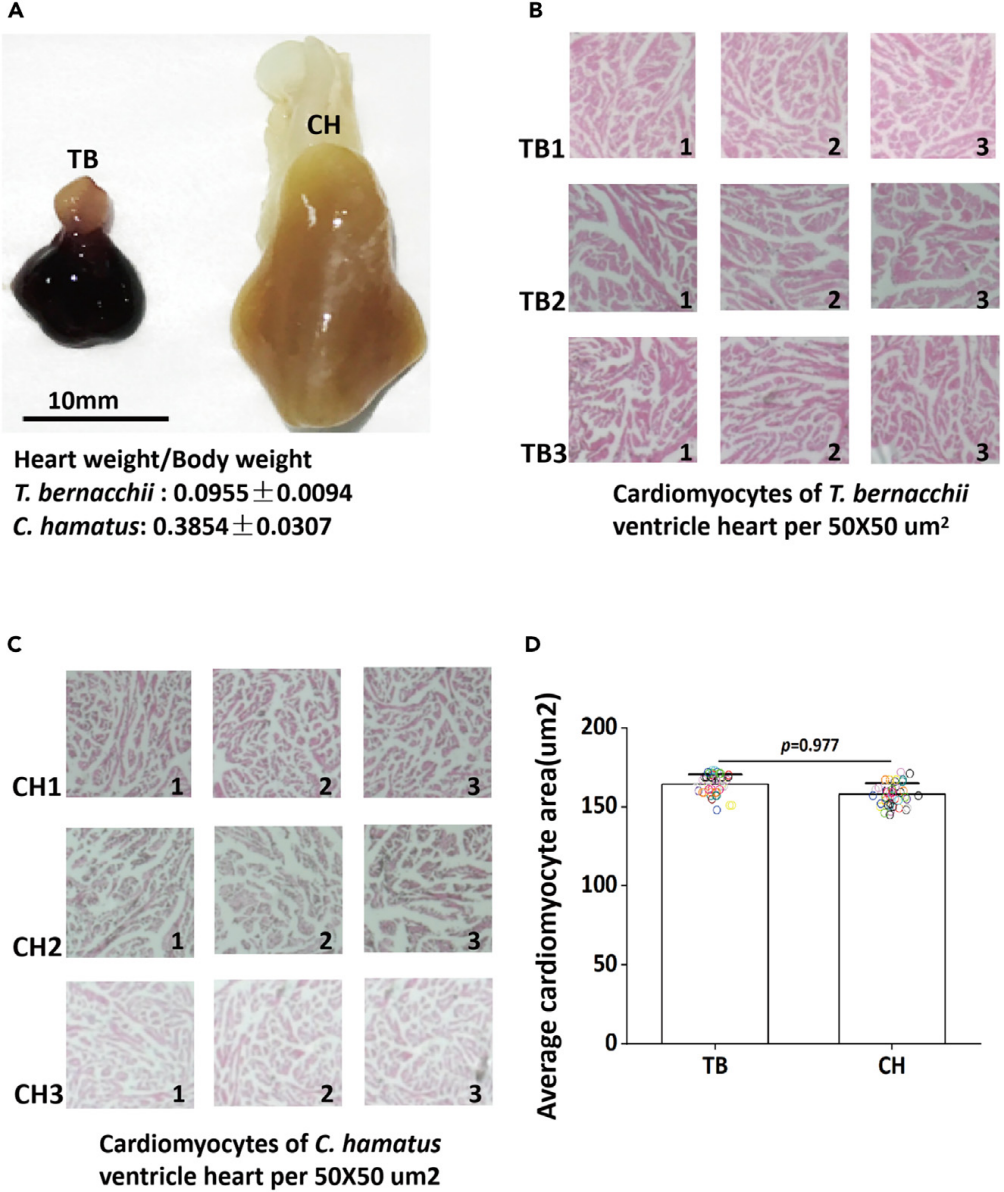
Morphology comparison between the white-blooded *C. hamatus* and red-blooded notothenioids *T. bernacchii* (A) The heart size comparison between *T. bernacchii* and *C. hamatus.* The average heart-to-body mass ratio of ten white-blooded *C. hamatus* and red-blooded notothenioids *T. bernacchii* is 0.3854 ± 0.0307 and 0.0955 ± 0.0094, respectively. The detailed biological parameters for the two Antarctic fish species were shown in Table S1. (B and C) Hematoxylin-eosin-stained ventricle paraffin sections of the two Antarctic fish species showed similar morphology. Three different individuals for the *T. bernacchii* (named as TB1, TB2 and TB3) and *C. hamatus* (named as CH1, CH2 and CH3) were used and 3 randomly chosen square areas (50 × 50μm^2^) (marked as 1, 2 and 3) per one individual were used for comparison. (D) Cardiomyocytes measurement comparisons of *T. bernacchii* and *C. hamatus* (Error bars indicate ±1 standard error of the mean (SEM)).

The body mass, length, and heart-to-body mass ratio of ten individuals of *C*. *hamatus* and *T. bernacchii* were measured, respectively (Table S1), from which the average heart-to-body mass ratio of *C*. *hamatus* is calculated to be 0.3854 ± 0.0307, which is 4 times larger than that of *T. bernacchii* heart-to-body mass ratio (0.0955 ± 0.0094). There was a significant difference between the heart-to-body mass ratio of two fish (*p*-value < 0.000002). The large *C. hamatus* heart is in pinkish brown color due to the presence of myoglobin albeit complete absence of hemoglobin in contrast with the small dark red appearance of the *T. bernacchii* heart (Figure 1A)*.* Hematoxylin-eosin stained paraffin sections of ventricles from the two Antarctic fish species showed similar sponge-like organization of cardiomyocyte (Figures 1B and 1C). Cross-section sizes from randomly selected 50 cardiomyocytes of *C*. *hamatus* and *T. bernacchii*’s hearts were determined using ImageJ software (Table S2). It was obviously that the size of cardiomyocytes was similar in the two species (Figure 1D and Table S2). Therefore, the remarkably enlarged heart of the icefish is attributed to higher cardiomyocyte number rather than cardiac hypertrophy resultant of cell body enlargement.

### Differential cardiac gene expression between the white-blooded icefish and red-blooded notothenioids

To elucidate the genetic programs underpinning the enlarged heart in icefish, the *C. hamatus* (CH) heart transcriptome was then compared with those from two red-blooded notothenioids (*T. bernacchii* (TB) and *G. acuticeps* (GA)) using a cross-species transcriptome comparison pipeline developed previously.^26^ The number of raw and clean reads was detailed in Table S3. 982 genes with an absolute log2Ratio value ≥ 1 and exhibiting the same up- or down-regulation trend in the CH/GA and CH/TB comparisons were identified as differentially expressed genes (DEGs) between the white-blooded and red-blooded notothenioids. Of the DEGs, 531 were downregulated while 451 were upregulated in the icefish heart (Table S4).

**Figure 2:**
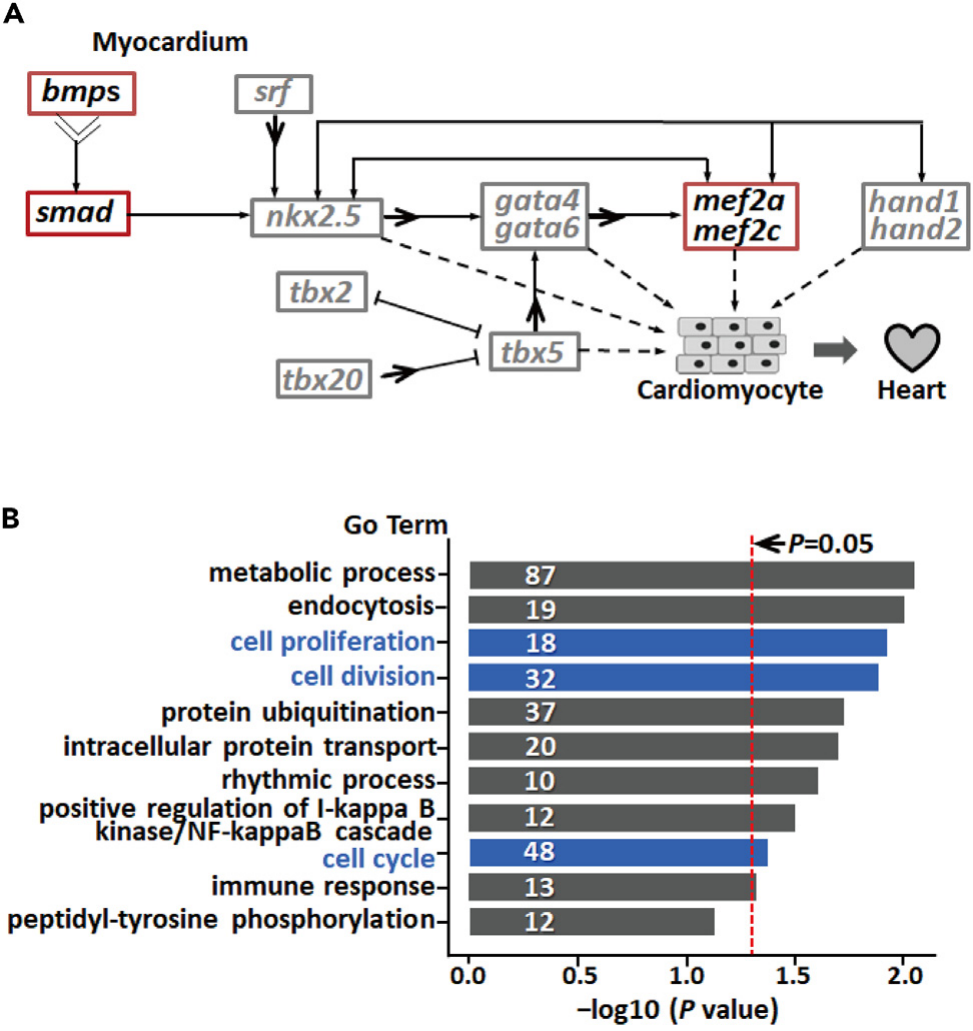
A schematic presentation of the regulatory networks involved in vertebrate heart development (A) The factors boxed in red rectangles were shown to be up-regulated in icefish heart compared with the red-blooded species *T. bernacchii* and *G. acuticeps*. These genes included *bmp2*, *bmp7*, *smad1*, *smad 2*, *smad 9*, *mef2a* and *mef2c* (Table S4). Factors boxed by gray rectangles showed similar transcription abundance between the hearts of two types of notothenioid fishes. The two-directional black arrows indicate mutual signaling between two TFs. The one-directional black arrow and dashed arrows indicate transcriptional activation or inhibition between the two factors, respectively. The dotted segments indicate non-definitively determined transcriptional signaling leading to cardiomyocyte’s differentiations. (B) Distribution of significantly enriched GO terms in the DEGs between the hearts of icefish and red-blooded notothenioids. Significant enrichment of 10 GO terms was found (*p* < 0.05, Number of genes involved in one GO term≥10). The GO terms involved in cell multiplication (cell proliferation, cell division, and cell cycle) are indicated by blue color. The X axis shows the –log10 *p* value of the GO enrichment. The red dotted segment indicates–log10 *p* = 0.05. And the Y axis shows the name of enriched GO terms.

The regulatory networks controlling cardiac development are highly conserved from fish to mammals and are depicted in Figure 2A(adapted from Bruneau 2013).^27^ All of the major factors were indeed found to be present in the heart transcriptomes of the three notothenioids, but with varying abundances (Table S5). While many of the regulators were expressed similarly between the icefish and red-blooded fishes, some of them were significantly upregulated in the icefish heart. These factors included several bone morphogenetic proteins (BMPs, BMP2, BMP7), the downstream proteins of BMP signaling (Smad1, Smad 2, and Smad 9), and members of MEF2 transcription factors (MEF2a, MEF2c) (Figure 2A and Table S5). BMPs and their downstream smad proteins, and MEF2s are indispensable for cardiomyocyte differentiation.^28^^,^^29^^,^^30^^,^^31^ Activation of *bmp2*/pSmad1/5/8 signaling pathways promotes cardiomyocyte proliferation in both adult and embryonic heart.^32^^,^^33^

We then performed GO enrichment tests on the DEGs, and found significant enrichment of 10 GO terms (*p* < 0.05, Number of genes involved in one GO term≥10), of which three (cell proliferation, cell division, and cell cycle) are involved with cell multiplication (Figure 2B). The majority of the genes associated with these three GO categories were upregulated in the icefish heart (Table S6), which included *cyclin d1 (ccnd1)*, *cyclin d2* (*ccnd2*), and c*yclin a2* (*ccna2*), known important regulators driving cardiomyocyte proliferation.^34^^,^^35^^,^^36^ The upregulation of the *bmps* and the cell division regulators in icefish heart in relative to the red-blooded fishes are verified by qRT-PCR (Table S7). Taken together, the transcriptome comparison suggests elevated cardiogenic poise through upregulation of cardiomyocyte differentiation and proliferation in icefish heart.

### Western blot analysis of BMPs and cardiac developmental markers in the hearts of *C. hamatus* and *T. bernacchii*

**Figure 3:**
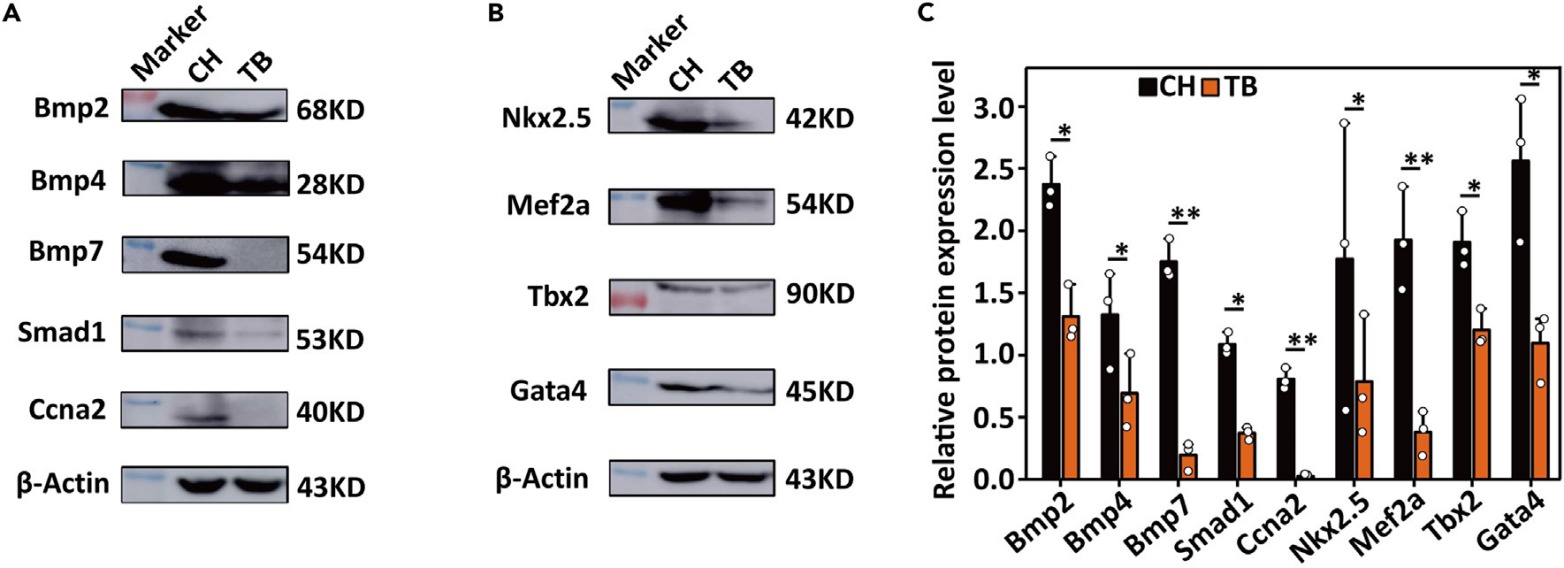
Western blot analyses of some cardiac developmental regulators in *C. hamatus* and *T. bernacchii* hearts (A and B) Results of western blot analyses of 10 factors involved in cardiac development in the hearts of *C. hamatus* and *T. bernacchii*. The range of the used protein molecular weight markers is 10–180 kD. The corresponding molecular marker sizes besides each detected protein are as follows: 75KD for Bmp2, 35KD for Bmp4, 60KD for Bmp7, 60KD for Smad1, 45KD for Ccna2, 45KD for Nkx2.5, 60KD for Mef2a, 75KD for Tbx2, 45KD for Gata4 and 45KD for β-actin. (C) The relative abundance of the examined proteins derived from three biological replicates (Error bars indicate ±1 SEM). Levels with significant difference between *C. hamatus* and *T. bernacchii* are denoted with ‘∗’ (*p* < 0.05, Student’s *t* test) and ‘∗∗’ (*p* < 0.01).

Based on the availability of suitable antibodies, we examined the abundance of the cardio-development regulators at the protein level. Compared with heart from red-blooded *T. bernacchii*, the icefish heart clearly presented greater abundance of Bmp2, Bmp4 and Bmp7 and the downstream protein Smad1 (Figure 3A), suggested upregulation of the BMP signaling in icefish heart. Consistently, substantial elevation of the downstream pro-cardiogenetic factors such as NK2 Homeobox 5 (Nkx2.5) and GATA binding protein 4 (Gata4) both essential for cardiac development,^37^^,^^38^^,^^39^^,^^40^ and Mef2a required for cytoarchitectural integrity in cardiac muscle development^41^ were detected in the icefish heart (Figures 3B and 3C). T-Box Transcription Factor 2 (Tbx2), a central intermediary of Bmp-Smad signaling facilitates endocardial cushion formation was also elevated.^42^

Progression through the cardiac cell cycle is tightly regulated and involves Cyclins complexed with their catalytic partners, the Cyclin-dependent kinases (Cdks).^42^ Cyclin A2 (Ccna2) complexed with Cdk2 is essential for the G1/S transition and Ccna2/Cdk1 promotes entry into mitosis.^43^ Ccna2, normally silenced in the postnatal heart, was reported to mediate cardiomyocyte mitosis in the postmitotic myocardium and induce cardiac enlargement when constitutively expressed from embryonic day 8 into adulthood ^44^ 1 Significantly upregulated expressions of Ccna2 were detected in the icefish heart (Figures 3A and 3C). The higher abundance of BMP morphogens, the downstream regulators, and Ccna2 suggests elevated cardiomyocyte multiplication (or hyperplasia) in the icefish heart.

### Differentially expressed miRNAs between the white-blooded and red-blooded fish hearts and target prediction

A total of 146 miRNAs were identified by sequencing the small RNA libraries derived from the hearts of the icefish and the two red-blooded species, and these were used for expression comparison. A total of 25 miRNAs (17.1%) exhibited ≥2.0-fold changes and presented the same up- or downregulation trend in the CH/GA and CH/TB comparison matches (Table 1). Among them, 14 miRNAs were reduced in the icefish heart (green column in Table 1), while 11 (red column in Table 1) were elevated. The reliability of the differentially expressed miRNAs between *C. hamatus* and *T. bernacchii* detected by the sequencing-based analysis was verified by quantitative RT-PCR on randomly selected miRNAs (Table S9).

**Table 1:** Differentially expressed miRNAs in the hearts of *C. hamatus*, *G. acuticeps* and *T. bernacchii.*

Rep miRNA ID	Rep Sequence	CH CPM∗	GA CPM∗	TB CPM∗	log2(CH/GA)	log2(CH/TB)
gmo-miR-451-5p	AAACCGUUACCAUUACUGAUU	78	104923	10284	−9.391	−7.075
gmo-miR-2188-3p	GCUGUGUGAGGUCGGACCUAUC	2	894	47	−7.877	−4.654
ola-miR-144-5p	AGGAUAUCAUCUUAUACUGUAAGU	1	367	138	−7.467	−7.089
gmo-miR-2188-5p	AAGGUCCAACCUCACAUGUCCU	14	2231	807	−6.334	−5.901
gmo-miR-143-3p	UGAGAUGAAGCACUGUAGCU	525	48802	1188	−5.527	−1.201
gmo-miR-144-3p	CUACAGUAUAGAUGAUGUACUAU	9	297	346	−3.990	−5.247
gmo-miR-181-5p	AACAUUCAUUGCUGUCGGUGGG	80	1752	201	−3.440	−1.353
gmo-miR-27b-3p	UUCACAGUGGCUAAGUUCUG	221	4820	587	−3.433	−1.431
gmo-miR-182-5p	UUUGGCAAUGGUAGAACUCACU	1	17	16	−3.000	−3.939
oni-miR-147	GUGUGCGGAAAUGCUUCUGCUC	4	37	68	−2.229	−4.135
gmo-miR-10c-5p	UACCCUGUAGAUCCGGAUUUGU	72	659	204	−2.186	−1.526
gmo-miR-724-5p	UUAAAGGGAAUUUGCGACUGUU	7	58	28	−2.134	−2.110
gmo-miR-204-5p	UUCCCUUUGUCAUCCUAUGCCU	14	122	67	−2.086	−2.269
oni-miR-458-3p	AUAGCUCUUUAAAUGGUACUG	28	39	607	−1.047	−3.044
oni-miR-1388	AGGACUGUCCUACCUGAGAAUG	199	198	66	1.015	1.573
gmo-miR-17-5p	CAAAGUGCUUACAGUGCAGGUA	83	83	27	1.019	1.588
oni-miR-15c	CAAAUCAUUUUGUGCUGCCACC	22	21	3	1.036	2.667
gmo-miR-133a-3p	UUGGUCCCCUUCAACCAGCUGU	5146	4666	2435	1.152	1.056
gmo-miR-30c-5p	UGUAAACAUCCUACACUCAGCU	496	428	139	1.222	1.809
dre-miR-22a-5p	AGUUCUUCACUGGCAAGCUUU	76	59	35	1.374	1.101
gmo-miR-20a-5p	UAAAGUGCUUAUAGUGCAGGUAG	70	51	23	1.452	1.599
dre-miR-29b-3p	UAGCACCAUUUGAAAUCAGUG	38	28	16	1.463	1.277
oni-miR-133b	UUUGGUCCCCUUCAACCAGCU	115	81	51	1.515	1.145
gmo-miR-190a-2-5p	UGAUAUGUUUGAUAUAUUAGGUU	19	13	2	1.516	3.094
gmo-miR-223a-3p	UGUCAGUUUGUCAAAUACCCCA	242	163	34	1.575	2.802

Note: MicroRNAs underlined indicate the microRNAs shared by the hypoxia acclimated zebrafish heart.

CPM∗ indicates the Counts per million. Latin names in italics.

As revealed by transcriptome analysis and western blot analysis, all of the seven cardiac developmental marker genes we tested were upregulated (Figures 2 and 3), therefore, we focused on the 14 miRNAs that showed downregulation in the icefish heart (green column in Table 1) to predict their possible targets. Using the 3′UTRs of *bmp2*, *mef2a*, *mef2c*, *gata4*, GATA binding protein 6 (*gata6*), *tbx2*, T-Box Transcription Factor 20 (*tbx20*) and Heart And Neural Crest Derivatives Expressed 2 (*hand2*) obtained from transcriptome sequencing, we were able to identify that all of the 14 downregulated miRNAs are potential regulators of the eight cardiac developmental marker genes (Table S10), suggesting possible functions of these miRNAs in heart enlargement.

### Screening for the microRNAs involved in heart enlargement

**Figure 4:**
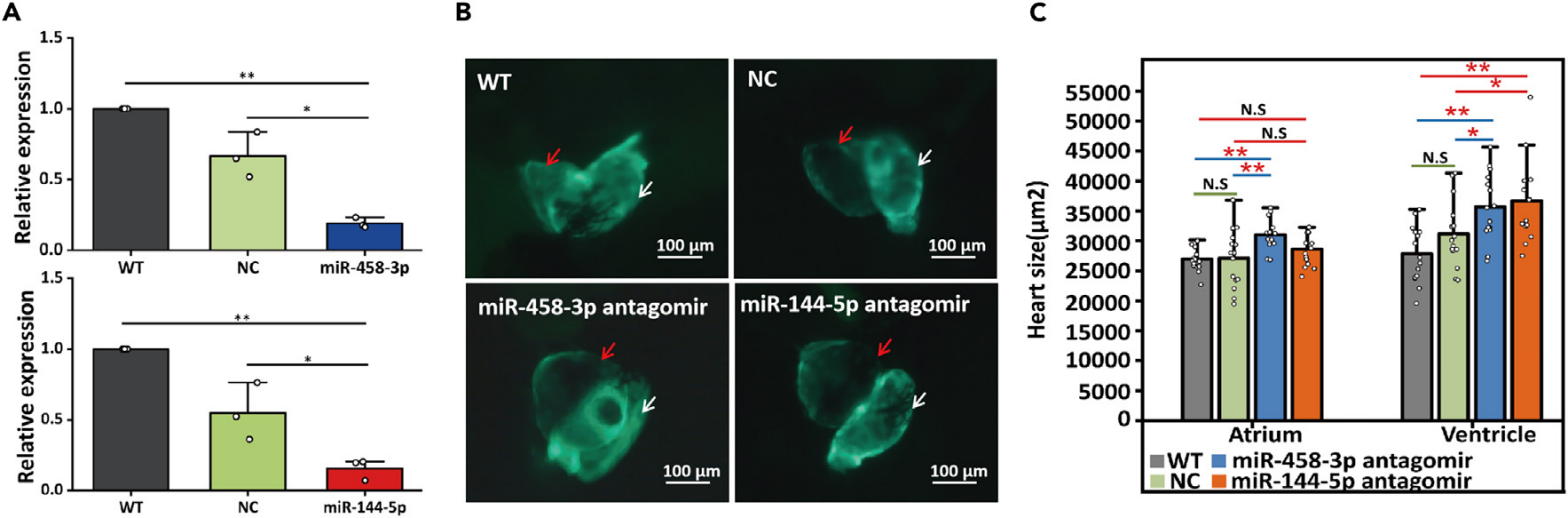
Experimental verifications of the regulatory functions of miR-458-3p and miR-144-5p on cardiomyocyte proliferation (A) Verification of the expression of miR-458-3p (upper)/miR-144-5p (down) in the embryo fish heart micro-injected by miR-458-3p antagomir (upper)/miR-144-5p antagomir (down). MicroRNA qRT-PCR techniques showed the significant down-regulation of miR-458-3p (or miR-144-5p) in the zebrafish heart micro-injected by miR-458-3p antagomir (or miR-144-5p antagomir). At least three biological replicates were conducted for each measurement. Treatments with significant difference (*p* < 0.05, *p* < 0.01, Student’s *t* test) were denoted with ‘∗’ and ‘∗∗’, respectively. (B) Tg *Danio rerio* (myl7: EGFP) were used for the microinjection experiment. Images of zebrafish development at 72 hpf after microinjection of 50 nM synthetic miR-458-3p antagomir (miR-144-5p antagomir) or NC, compared with the WT zebrafish showing the different heart sizes. The white arrow and red arrow indicate atrium and ventricle, respectively. (C) Comparison of heart sizes measured from each treatment. Treatments with significant difference (*p* < 0.05, *p* < 0.01, *p* < 0.001, Student’s *t* test) were denoted with ‘∗’, ‘∗∗’ and ‘∗∗∗”, respectively. N.S. is the abbreviation for none significant. Error bars indicate ±1 SEM.

To screen for miRNAs that function in heart enlargement, we synthesized antagomirs of the 14 miRNAs and individually microinjected into zebrafish embryos. The heart size including atrium and ventricle of the developing embryos at 72 hpf were measured and compared with those of individuals microinjected with negative control (NC) miRNA or untreated individuals (WT) at the same age. Out of the 14 microRNA antagomirs, except 2 miRNA antagomirs (miR-458-3p and miR-144-5p), 12 miRNA antagomirs showed no effects regarding to heart enlargement (detailed results omitted). As shown on Figure 4, the miR-458-3p antagomir and miR-144-5p antagomir resulted in significantly enlarged heart in the injected fishes (Figure 4B). The average sizes of atrium and ventricle of the embryos micro-injected by the miR-458-3p antagomir were approximately 15% and 14.5% larger than the individuals from the NC groups (*p* = 0.0095∗∗ and *p* = 0.036∗, respectively, two-tailed *t*-test) (Figure 4C and Table S11). More significant enlargement (approximately 18% larger) was seen in the ventricles of the fishes injected with the miR-144-5p antagomir when compared with the NC group fishes (*p* = 0.0227∗, two-tailed t-test) (Figure 4C and Table S11). Those statistics were also significant when compared with fishes of the untreated (WT) group. No other miRNA antagomirs were found to have significant effects on heart size in this *in vivio* screening.

In order to ensure that the administration of the two antagomirs had suppressed the expression of their targeted miRNAs, we compared the expression levels of miR-458-3p and miR-144-5p in the zebrafish hearts from the antagomir, NC and WT groups by qRT-PCR, and positive results were obtained (Figure 4A and Table S12).

### Verification of the miR-458-3p and miRNA-144-5p antagomirs on cardiomyocytes proliferations

**Figure 5:**
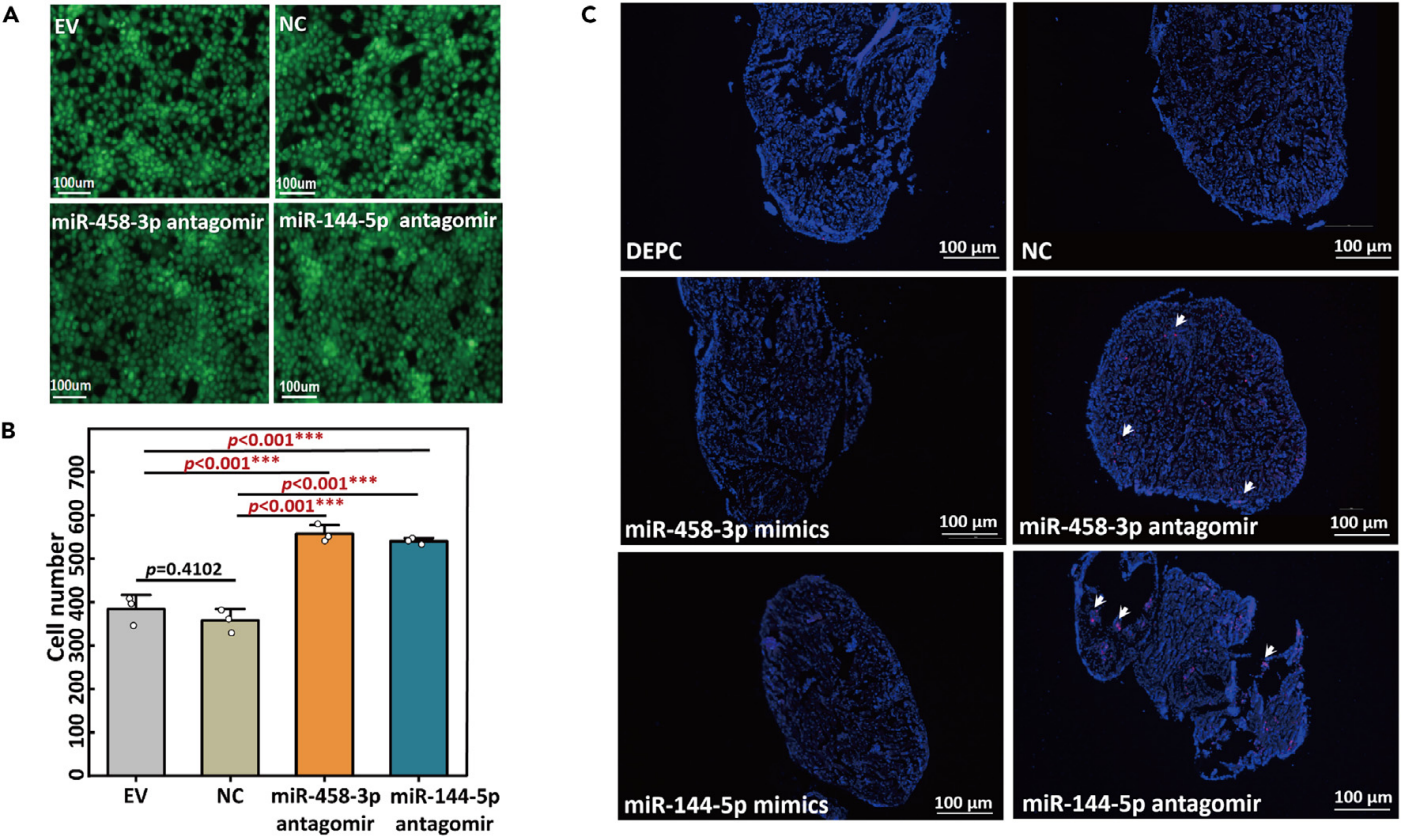
Verification of the miR-458-3p and miRNA-144-5p antagomirs on cardiomyocytes proliferations (A) Images of equal numbers of cultured H9C2 cardiomyocytes transfected with miR-458-3p antagomir, miR-144-5p, negative control (NC), and empty vector (EV), respectively. (B) Cell number counting and statistical analysis for the transfected cells. Treatments with significant differences (*p* < 0.001, Student’s *t* test) are indicated by ‘∗∗∗’ (Error bars indicate ±1 SEM). (C) The apex cordis excisional zebrafish microinjected by DEPC water, NC miRNA, miR-458-3p mimics and miR-144-5p mimics showed no significant proliferation signals. The apex cordis excisional zebrafish microinjected by miR-458-3p antagomir or miR-144-5p antagomir showed a significant proliferation signal (red dot).

To see whether miR-458-3p and miR-144-5p down-regulation would have effects on cardiac development, we transfected the H9C2 cells with a miR-458-3p antagomir, a miR-144-5p antagomir or an NC miRNA, and the efficiency of cell proliferation was measured after 48 h. The total cell number in the group transfected with miR-458-3p antagomir (or miR-144-5p antagomir) was about 1.4-fold higher than the empty vector (EV) and control groups (∗∗∗*p* < 0.001; Figures 5A and 5B). It was clear shown that the downregulation of miR-458-3p and miR-144-5p promoted the proliferation of the cardiac cells.

To further identify the functions of miR-458-3p and miR-144-5p on cardiomyocytes proliferations, we synthesized antagomirs of the two miRNAs and individually microinjected into the abdomen of the adult zebrafish with 15% apex cordis excision. Meanwhile, mimics of miR-458-3p and miR-144-5p, negative control (NC) miRNA and DEPC water were microinjected into the abdomen of the adult zebrafish with 15% apex cordis excision as controls. The paraffin sections of the zebrafish hearts by the different microinjections showed that apex cordis excisional zebrafish microinjected by miR-458-3p antagomir or miR-144-5p antagomir showed significant cell proliferation signals (red dot in Figure 5C) in contrast with those microinjected with DEPC water, NC miRNA, miR-458-3p mimics and miR-144-5p mimics (Figure 5C), indicating that down regulation of miR-458-3p or miR-144-5p in cardiomyocyte would promote cardiomyocytes proliferation.

### Verification of miR-458-3p and miR-144-5p target genes

**Figure 6:**
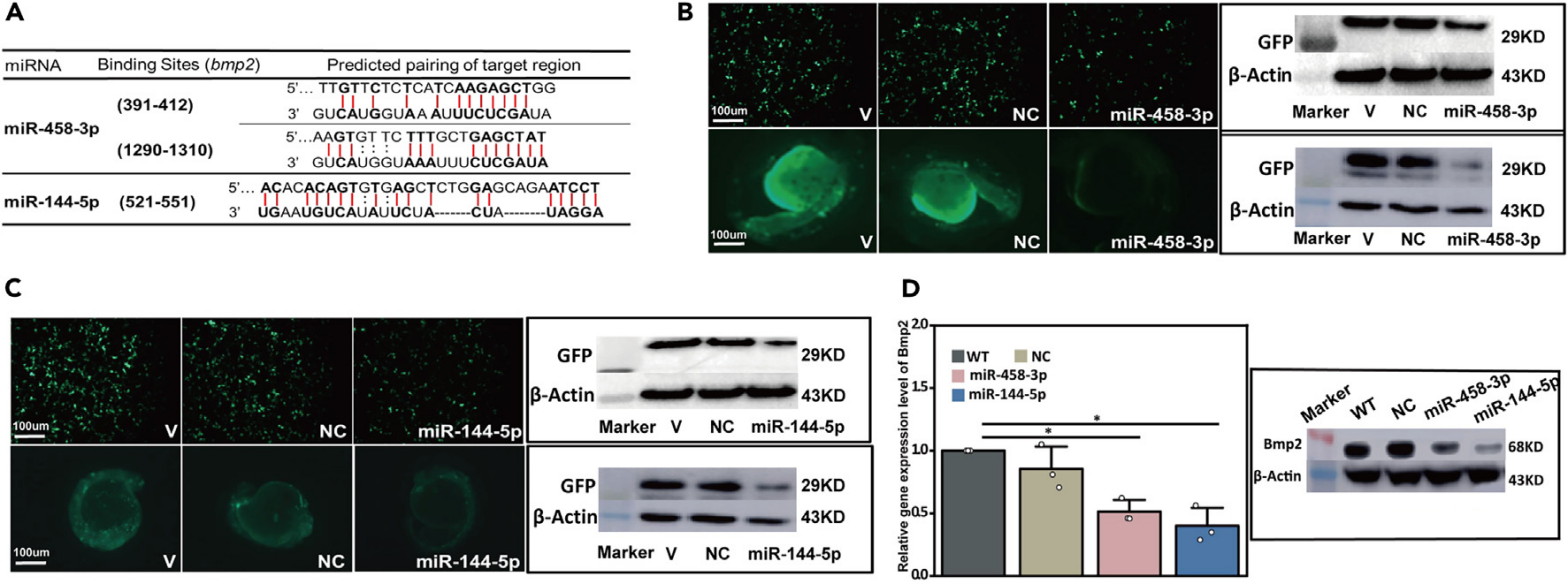
Experimental verifications of target genes of miR-458-3p and miR-144-5p (A) Predicted binding site of miR-458-3p at the 3′UTR of *bmp2* (The 3′UTR sequence of *bmp2* was shown in Table S8) and binding site of miR-144-5p at 3′UTR of *bmp2*. (B) Verification of the function of miR-458-3p on 3′UTR of *bmp2* using EGFP reporter constructs in the H9C2 cardiomyocyte cell line and zebrafish fertilized 1- to 2-cell embryos, respectively. Compared to the cells transfected with vector (V), the NC microRNA, the cells transfected with the miR-458-3p mimics showed significantly reduced EGFP expression by the pEGFP-*bmp2*-3′UTR construct. The expression of GFP was measured using Western blotting after 48 h post transfection. (C) Verification of the function of miR-144-5p on 3′UTR of *bmp2* using EGFP reporter assay. (D) *bmp2* expression comparisons between WT, zebrafish embryos microinjected by NC miRNA, miR-458-3p antagomir, and miR-144-5p antagomir by qRT-PCR and western blotting analysis. Treatments with significant differences (*p* < 0.05, Student’s *t* test) are indicated by ‘∗’ (Error bars indicate ±1 SEM). The range of the used protein molecular weight markers is 10–180 kD. The corresponding molecular marker sizes besides each detected protein are as follows: 75KD for Bmp2, 29KD for GFP and 45KD for β-actin.

By using the 3′UTRs of *bmp2*, *mef2a*, *mef2c*, *gata4*, *gata6*, *tbx2*, *tbx20*, and *hand2* obtained from transcriptome sequencing of the icefish, we conducted target gene prediction. Among the eight cardiac developmental marker genes, miR-458-3p was found to solely target *bmp2*, while mR-144-5p targeted 6 genes (Table S10), including *bmp2* (Figure 6A). Similarly, miR-458-3p and miR-144-5p could target *bmp2* in zebrafish.

We then experimentally verified the suppression effects of miR-458-3p and miR-144-5p on *bmp2* expression. We constructed vectors containing the EGFP coding sequence linked with the 3′ UTR of the *bmp2* gene (Figure S2) and co-transfected the vector with either the miR-458-3p mimics or miR-144-5p mimics into cells of a cardiomyocyte cell line H9C2 or zebrafish embryos. Compared with the controls, the expression of GFP was markedly reduced in both in the cardiomyocyte cell line and the zebrafish embryos containing the miR-458-3p mimics or miR-144-5p mimics (Figures 6B and 6C). Thus, both miR-458-3p and miR-144-5p could suppress *bmp2* expression in both the cardiac *cell line* and *in vitro* (embryo) conditions.

Given the fact that miR-458-3p and miR-144-5p target *bmp2*, we then tested whether the microinjection of the mimics of the two miRNAs in zebrafish embryos would downregulate the expression levels of *bmp2*. Both qRT-PCR and western blot results supported the hypothesis (Figure 6D).

### A common phenomenon of hypoxia induced profile change of the cardiogenic factors in fishes

**Figure 7:**
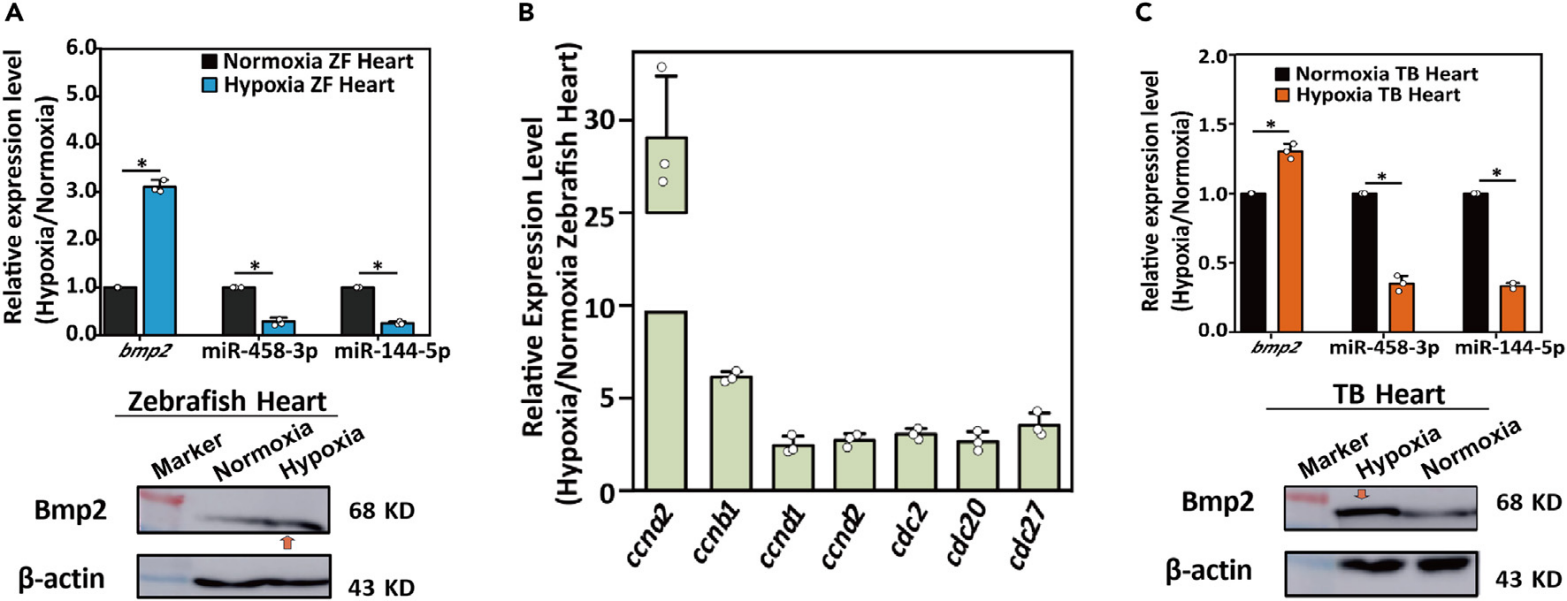
Hypoxia induced miR-458-3p/miR-144-5p downregulation and *bmp2* upregulation in zebrafish heart and Antarctic fish heart (A) Quantitative RT-PCR showing up-regulated expression of *bmp2*, reduced miR-458-3p and miR-144-5p in the hypoxia (DO = 1.0 ± 0.2 mg/L) acclimated zebrafishes’ heart (see Table S14 for details). Western blot analysis of Bmp2 in the DO = 1.0 ± 0.2 mg/L acclimated zebrafishes’ heart compared with that of the normoxia zebrafishes. (B) Quantitative RT-PCR showing upregulated expressions for the cell cycle-related genes *cdc2*, *cdc20*, *cdc27*, *ccnd1*, *ccnd2*, *ccna2* and *ccnb1* in the heart of hypoxia acclimated zebrafish (see Table S15 for details). At least three biological replicates were conducted for each measurement. (C) Quantitative RT-PCR showing reduced expression of *bmp2* and miR-458-3p in the conditioned red-blooded *T. bernacchii*s’ heart (see Table S16 for details). Western blot analysis of Bmp2 in the hypoxia conditioned red-blooded *T. bernacchii*s’ heart compared with that of the normoxia *T. bernacchii*. The relative abundance of the examined (*bmp2*) and the gene of miR-458-3p derived from at least three biological replicates. Levels with significant difference between the hypoxia conditioned red-blooded *T. bernacchii*s’ heart and that of the normoxia *T. bernacchii* are denoted with ‘∗’ (*p* < 0.05, Student’s *t* test). Error bars indicate ±1 SEM. The range of the used protein molecular weight markers is 10–180 kD. The corresponding molecular marker sizes besides each detected protein are as follows: 75KD for Bmp2 and 45KD for β-actin.

We reasoned that the drastic reduction of oxygen-carrying capacity in the hemoglobinless icefish could have resulted in hypoxic conditions in icefish tissues and organs, which might serve as driving force for the remodeling of the cardiovascular system in evolution and such processes might be partially simulated by model fishes exposed to a long-term hypoxic condition. We characterized and compared the microRNAomes of the hearts of zebrafishes exposed to 2–3 weeks of hypoxic (DO = 1.0 ± 0.2 mg/L, dissolved oxygen, DO) and normoxic (DO = 6.5 ± 0.2 mg/L) conditions. We identified 82 miRNAs and 57 miRNAs were down- and up-regulated in the hypoxic heart respectively (Table S13). Out of these miRNAs, 10 and 7 were consistent with the down- and up-regulated miRNAs observed in the icefish heart. In other words, among the 14 down-regulated miRNAs in the *C. hamatus* heart, 10 miRNAs were detected in the hypoxic zebrafish heart (See Table 1, the microRNAs underlined). More remarkably, the two miRNAs, miR-458-3p and miR-144-5p were also downregulated as seen in the hypoxic challenged zebrafish heart (Tables 1 and S13, Figures S3 and S4). The down-expressed miR-458-3p and miR-144-5p in the hypoxic challenged zebrafish heart was further verified by the mcrioRNA qRT-PCR (Figure 7A and Table S14). In addition, the hypoxic zebrafish recapitulated the upregulation of *bmp2* mRNA (Figure 7A) and the translated protein as well (Figure 7A). Such an increase was also detected for the downstream genes *cdc2*, *cdc20*, *cdc27*, *ccnd1*, *ccnd2*, *ccna2* and *ccnb1* controlling the cell cycle in heart (Figure 7B and Table S15). The fact that more than 50% of the hypoxic inducible miRNAs including the two cardiac enlargement-related miRNAs and their targeted genes are in consistent with the changing miRNA and target gene profiles found in the icefish heart supports our hypothesis of a hypoxia-driven scenario of cardiovascular remodeling in icefish. To further support this hypothesis, miR-458-3p, miR-144-5p and *bmp2* were found to behave in the similar manner when exposed to hypoxic condition in the Antarctic notothenioid, *T. bernacchii*, which is evolutionarily close related to the icefish and inhabits the same freezing environment as the icefish *C. hamatus* (Figure 7C and Table S16).

**Figure 8:**
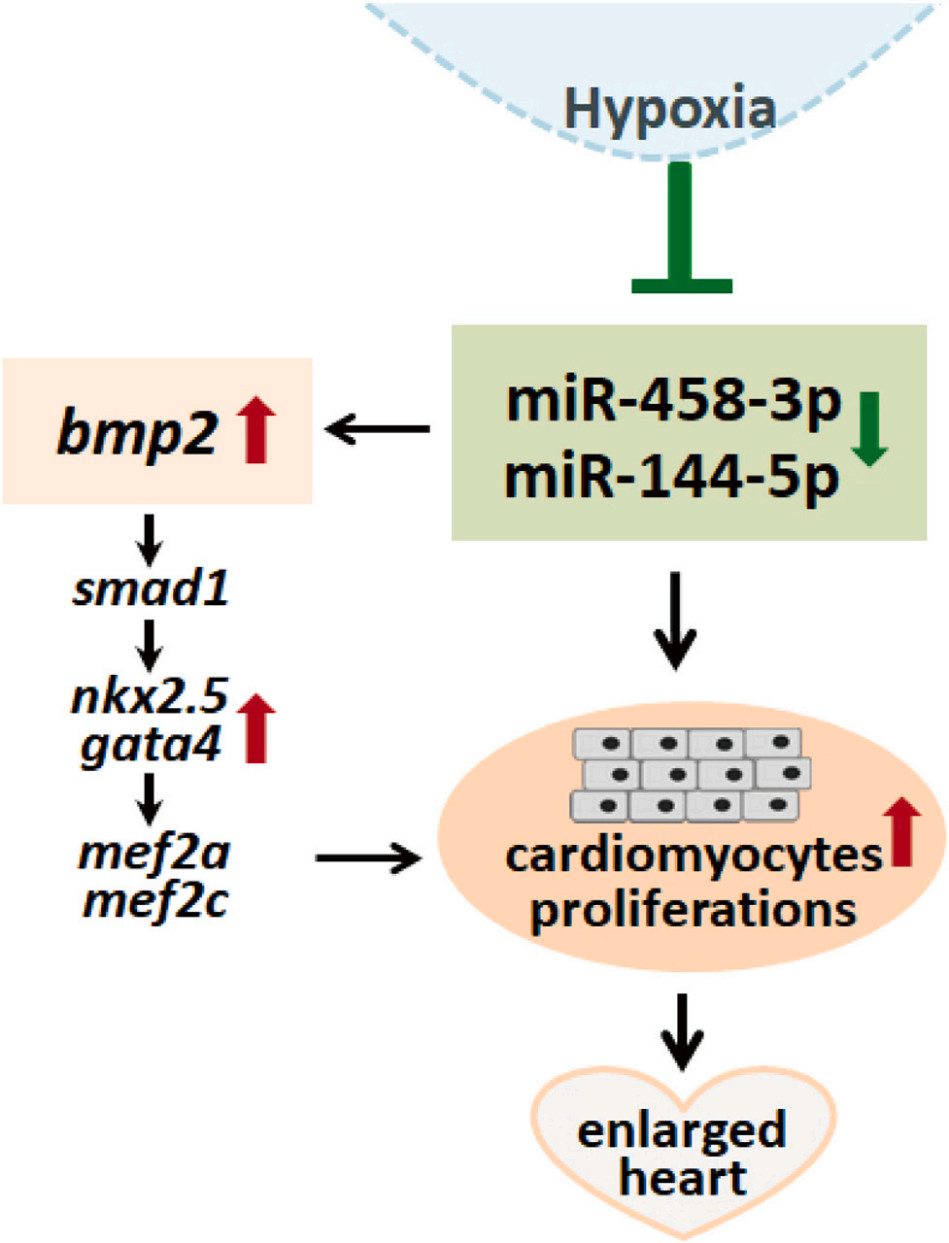
A proposed regulatory network for the adaptive heart enlargement in icefish

Taking this evidence together, we propose here a regulatory network for adaptive heart enlargement in icefish (Figure 8). In this network, hypoxia condition mediated the downregulation of the miR-458 and miR-144-5p, which result in the BMP2 upregulation accompanied by elevation of the downstream pro-cardiogenesis transcription factors function synergistically to promote cardiomyocyte proliferation. The high coincidence between the hypoxia-induced changes of gene expression profiles in zebrafish and those detected in the icefish suggested that the signaling pathways of heart remodeling upon hypoxia challenge in the two fishes are conserved.

## Discussion

In this study, we revealed signaling pathways that play roles in the adaptive size enlargement of the icefish heart. The enlarged icefish heart represents a key evolutionary adaptation for enhancing oxygen transport.^15^^,^^16^ Previous studies indicated that the proliferation of mitochondrial membranes in icefish provides the best pathway for the diffusion of intracellular oxygen.^45^ At lower temperatures, the membrane fluidity and structural integrity of icefish are damaged compared to the red-blooded notothenioids.^44^ The icefish heart also accommodates higher maximum stroke volume (VS) and maximum.^46^ In our study, we found the signaling pathways involved in icefish heart enlargement were substantially different from responses noted in cardiac hypertrophy in humans and other mammals occurred in response to pathological and physiological hemodynamic overload,^47^^,^^48^ in which heart size increases by cardiomyocyte enlargement without changing myocyte number.^49^ Instead of cardiac hypertrophy, the primarily cause of heart enlargement in the icefish was elevation of cardiomyocyte proliferation, hyperplasia in relative with the red-blooded Antarctic notothenioids.

A plethora of evolutionarily conserved signaling pathways and transcription factors regulating cardiomyocyte differentiation and proliferation have been uncovered in the vertebrates. Hedgehog, bone morphogenetic proteins (BMPs), fibroblast growth factors (FGFs) and non-canonical Wnt/JNK are known procardiogenetic factors expressed in endoderm and mesoderm during development.^50^ After specification of cardiac mesoderm, BMPs seem to be important for expansion of cardiac progenitors^51^ and for maintaining the balance between precursor expansion and differentiation.^52^ Cardiac-enriched genes, most importantly NKX2-5, SRF, GATA, T-box, and MEF2s transcription factors are later expressed in a tightly controlled cascade, which promotes cardiomyocyte differentiation and proliferation.^27^ In addition, numerous studies indicate that cell cycle regulators (e.g., CCNDs), Hippo signaling, Neuregulin signaling, microRNAs and hypoxia all play important roles in regulation of cardiomyocyte proliferation.^53^

A number of miRNAs have been demonstrated to be involved in cardiac morphology, development, function and pathogenesis of cardiovascular disease.^54^^,^^55^ We reasoned that miRNAs might play regulatory roles in regulating the essential cardiac developmental regulators that have shown altered expression in previous studies. To test this possibility, we studied the difference in the heart miRNA profiles between icefish and red-blooded fish. In this study, we identified two miRNAs, miR-458-3p and miR-144-5p that regulate heart size in fish. Knowledge on the function of miR-458-3p is so far very limited. It is reported that miR-458-3p is downregulated in the liver of avian leukosis virus-infected chicken,^56^ and in the brain of cold-acclimated zebrafish.^57^ Functional Studies on miR-144s are far more extensive. MiR-144-3p and miR-144-5p have been widely studied in numerous cancer types as tumor suppressors.^58^^,^^59^ While miR-144-3p has been reported to be a key factor regulating erythroid differentiation,^60^ and a cardioprotective agent in ischemic reperfusion.^61^^,^^62^ The function of miR-144-5p in cardiovascular development is not reported. This work is the first to discover the function of miR-458-3p and miR-144-5p in regulating cardiac development.

One of the mediators in the signaling cascade could be *bmp2* (Figure 2). *Bmp2*, a member of the TGF-β superfamily, play pivotal function in embryonic heart development.^34^^,^^63^ *Bmp2* expression in the endocardium is required for atrioventricular endocardial cushion maturation and remodeling^64^ and restricted myocardial *bmp2* expression is a key patterning signal for atrioventricular canal specification and valves formation.^65^ Over-expression of *bmp2* in myocardium maintains ventricular myocardium and cardiac progenitors in a primitive, proliferative state, indicating its role in the expansion of immature cardiomyocytes.^66^ Besides its function in embryonic cardiac development, *bmp2* is recently found to be essential for cardiomyocyte proliferation during heart regeneration in adult zebrafish.^66^ The functions of *bmp2* in promoting the expansion of immature cardiomyocyte during early development and cardiomyocyte proliferation during adult heart regeneration is consistent with its role as the downstream regulator of the miR-458-3p and miR-144-5p in heart size enlargement in fishes.

Evolutionarily, both miR-458-3p and miR-144s are vertebrate-specific,^67^ while miR-458-3p is present in fishes, reptiles, chicken and monotremes,^68^ miR-144s are found in all lineages of the vertebrates including the eutherian mammals (www.miRbase.org.version 22). The sequence alignments of miR-458-3p, miR-144-5p, and the sequences of the two miRNAs' binding cites in the *bmp2* gene demonstrate the evolutionary relationships among three Antarctic fish, which also have a relatively conservative relationship with the model organism, the zebrafish (Figure S5). Therefore, in this study, we used zebrafish as model to study the cardiogenic regulation function of both miRNAs *in vivo*. The differences between zebrafish and Antarctic fish are significant. However, due to the difficulty of capture, slow development times, long lives (about 30 years) and a lack of breeding colonies of the Antarctic fish species, we can only use zebrafish as the experiment model to verify the functions of the two miRNAs.

On the other hand, microRNAs usually target hundreds of genes, for example, miR-144-5p in this study is also demonstrated to suppress several important regulators (such as *mef2a*, *mef2c*, *gata4*, *gata6*, and *tbx2*) involved in cardiac development, besides *bmp2* (Table S10). Whether this interaction had resulted in the slightly higher activity of this miRNA in promoting heart enlargement than miR-458-3p detected in the experiment (Figure 4) could be future question to investigate. Furthermore, future studies on the differential responses of the factors in the BMP signaling in the cardiomyocytes of the adult mammalian and zebrafish hearts may shed new light on the mechanisms underlying the different regenerative capacities between the two.

An interesting finding in this study is that expression of both miR-458-3p and miR-144-5p are significantly downregulated under hypoxic condition in fish heart. The finding of hypoxia induced downregulation of miR-458-3p expanded its environmental responsive spectra from the known ones i.e., low temperature^57^ and in virus infection.^56^ Remarkably, hypoxia was found to significantly reduce miR-144 expression in human prostate cancer cells,^69^ and in mice myocardial tissue after myocardial infraction.^70^ In the latter case, hypoxia induction of an miR-144 antagonistic long noncoding RNA, metastasis-associated lung adenocarcinoma transcript 1 (MALAT1) was implicated in miR-144-3p reduction and reduction of miR-144-3p led to cardiomyocyte apoptosis.^70^ However, such studies have not been specifically focused on miR-144-5p. Nevertheless, it appears that both miR-458-3p and miR-144-5p are inducible by abiotic stresses, and this is property probably conserved in different animal lineages. Indeed, positive effects of hypoxia on heart regeneration have been reported in adult mice and zebrafish.^71^^,^^72^ It was found that mice exposed to hypoxia one week after induction of myocardial infarction induced robust regeneration by reactivation of cardiomyocyte proliferation. As for fish, fish under hypoxia environment (DO = 1.5 ± 0.2 mg/L, 2–3 months) would have significantly larger heart when compared with the nomoxic (DO = 6.5 ± 0.2 mg/L) cultured fish (unpublished data). The DOL (Dissolved Oxygen Level, DOL) in the hypoxia group was 1.5 ± 0.2 mg/L, which was based on previous hypoxia studies in zebrafish.^73^ Enlarged hearts after hypoxia acclimation was also detected in other fish species.^74^ Deciphering the mechanisms underlying the hypoxic responsiveness of these miRNAs and the cellular consequences will help us understanding cardiac development and heart regeneration.

In a previous study, we compared the microRNAome of the head kidney between the icefish *C. hamatus* and the red-blooded *T. bernacchii*, and found upregulation of more than hundred of miRNAs in the icefish while only five miRNAs are downregulated.^41^ The broad upregulation of miRNAs in this tissue is partially attributable to significant upregulation of TGF-β signaling in the icefish.^41^ Among those upregulated miRNAs, miR-152, miR-1388 and miR-16b^40^^,^^41^ are turned out to exert suppressive functions on the formation of erythroids. In contrast, no significant discrepancy is seen in the number of up- (11) and downregulated (14) miRNAs in the heart between the two species and the total number of differential expressed miRNAs in the heart is much fewer, suggesting tissue-specific regulations of miRNA biogenesis between the two tissues. Nevertheless, both studies indicated that miRNAs played important roles in developmental adaptation of the Antarctic icefishes to the hemoglobinless life. Specifically, the loss of hemoglobin and active erythrocytes in the icefish presumably produced a chronic hypoxic condition in the icefish tissues which altered the expression profiles of many miRNAs. Out of which, the reduction of miR-458-3p and miR-144-5p might have contributed to reprograming of cardiac development.

Despite advances in the prevention and treatment of cardiovascular diseases, heart failure remains one of leading cause of human mortality. Heart failure often results from cardiomyocyte loss, and heart regeneration aimed to replace the lost cardiomyocytes is a field of intensive study.^31^ It is now accepted that the adult mammalian heart undergoes a low grade of cardiomyocyte turnover which can be augmented by activating the redeployment of pro-cardiogenic factors.^75^ Mechanisms for cardiac development are highly conserved from fish to man, and indeed we found highly similar factors that were involved in cardiomyocyte proliferation between the icefish and the mammals in this study. Therefore, the adult icefish heart might serve as a model to study the factors that regulate heart regeneration. Whether the miRNAs identified in this study may function in cardiomyocyte proliferation in mammals is an interesting question for further study.

To summarize, we found the exceptionally large heart of the Antarctic icefish is a result of elevated hyperplasia compared with its red-blooded relatives. This developmental adaptation is found to be associated with substantial shifts in both the transcriptomes and the miRNAome in the icefish heart. Especially, we identified two miRNAs, miR-458-3p and miR-144-5p, work as important regulators in cardiomyocyte proliferation through targeting the BMP2 pathways of heart development. We further revealed the miRNAs-BMP2 signaling cascade is likely induced by the hypoxic condition in the icefish body.

### Limitations of the study

Due to the lack of live Antarctic icefish samples for conducting functional validation experiments, most of the functional validation experiments in this study are based on results obtained from the model organism zebrafish. This is the biggest limitation of this study, and it remains unresolved to date.

## STAR★Methods

### Key resources table

**Table:** 

REAGENT or RESOURCE	SOURCE	IDENTIFIER
**Antibodies**		

Nkx2.5	Santa Cruz Bio	sc-376565
Tbx 2	OriGene	TA344550
Gata4	GeneTex	Cat# GTX113194; RRID: AB_10726138
Bmp2	HuaAn Bio	ER80602
Bmp4	GeneTex	Cat# GTX128348; RRID: AB_2885760
Bmp7	Sigma	QC49491
Smad1/5/9	Abcam	ab66737
Mef2A	GeneTex	Cat# GTX50398; RRID: AB_11172776
Ccna2	LSBio	Cat# LS-C31034; RRID: AB_898104
β-actin	HuaAn Bio	M1210-2
GFP	GeneTex	GT859

**Critical commercial assays**		

RT-PCR kit	TaKaRa	RR014A
enhanced chemiluminescence (ECL) reagent kit	Millipore	407207
miRNeasy Mini kit	QIAGEN	217084
mirVanaTM miRNA Isolation kit	Ambion	AM1561
poly(A) polymerase	Ambion	AM2030
HiPerFect Transfection Reagent	QIAGEN	301704
Edu (5-Ethynyl-2′-deoxyuridine)	Guangzhou Shuopu Biotechnology Co., Ltd	CAS: 61135-33-9
Cell-Light EdU Apollo567 *In Vitro* Kit	Guangzhou Ribo Biotechnology Co., Ltd	C10310-1

**Deposited data**		

All sequencing data associated with this project	http://www.ncbi.nlm.nih.gov/bioproject/1069298	PRJNA1069298

**Experimental models: Cell lines**		

H9C2	College of Marine Sciences, Shanghai Ocean University	CSTR:19375.09.3101RATGNR5

**Experimental models: Organisms/strains**		

*C. hamatus*	Antarctica (74°55′S, 163°46′E) Antarctica (69°22′S, 76°22′E).	Ross Sea Prize Bay
*T. bernacchii*	Antarctica (74°55′S, 163°46′E) Antarctica (69°22′S, 76°22′E).	Ross Sea Prize Bay
*G. acuticeps*	Antarctica (69°22′S, 76°22′E).	Prize Bay
*Danio rerio*	China Zebrafish Resource Center	myl7: EGFP

**Oligonucleotides**		

miR-458-3p	5′CAGUACCAUUUAAAGAGCUAU3′	Shanghai Gene Pharma Co. Ltd.
miR-144-5p	5′ ACUUACAGUAUAAGAUGAUAUCCU 3′	Shanghai Gene Pharma Co. Ltd.

**Software and algorithms**		

Trinity	version 2.4.0	https://github.com/trinityrnaseq/trinityrnaseq/releases
SwissProt	Version 2022.10	http://www.expasy.ch/sprot
Blast2GO	Version 2.5	https://www.blast2go.com/
Bowtie	Version 2.4.1	https://bowtie-bio.sourceforge.net/bowtie2/index.shtml
common reference *Dissostichus mawsoni*	https://www.ncbi.nlm.nih.gov/bioproject/PRJNA401363/	PRJNA401363
DESeq2 package	version: 1.2.9	https://bioconductor.org/packages/release/bioc/html/DESeq2.html
miRNAs in miRbase	22.0.	https://www.mirbase.org/
DESeq	Version 1.18.0	https://bioconductor.org/packages//2.10/bioc/html/DESeq.html
Rfam	Version 14.8	http://sanger.ac.uk/software/Rfam
GenBank	Version 250	http://blast.ncbi.nlm.nih.gov
GraphPad Prism 5.0	GraphPad software	https://www.graphpad.com/support/prism-5-updates/
Image J	NIH	https://imagej.net/software/imagej/

### Resource availability

#### Lead contact

Further information and requests for resources and reagents should be directed to Liangbiao Chen (lbchen@shou.edu.cn).

#### Materials availability

This study did not generate new reagents.

#### Data and code availability

• All sequencing data associated with this project were deposited in the National Center for Biotechnology Information (NCBI) Sequence Read Archive database (BioProject Accession Number: PRJNA1069298). All the dataset is declared to be publicly accessible.
• This paper does not report original code.
• Any additional information required to reanalyze the data reported in this paper is available from the lead contact upon request.

### Experimental model and study participant details

#### Ethics declaration

We followed the NIH guidelines for Antarctic fish and zebrafish euthanasia (https://oacu.oir.nih.gov/sites/default/files/uploads/arac-guidelines/zebrafish.pdf). All handling of fish was carried out in accordance with the guidelines on the care and use of animals for scientific purposes set up by the Institutional Animal Care and Use Committee (IACUC) of Shanghai Ocean University (SHOU), Shanghai, China. This research was approved by the IACUC (IACUC SHOU-DW-2012-002, SHOU-DW-20171022) of SHOU.

#### Fish

White-blooded *C. hamatus* and red-blooded notothenioids *T. bernacchii* were collected from Ross Sea, Antarctica (74°55′S, 163°46′E) with the water depth of 130-160m. Ten *C. hamatus* (5 males and 5 females) and ten *T. bernacchii* (5 males and 5 females) individuals were used for biological measurement including body mass and length measurements.

Three *C. hamatus* individuals (1 male and 2 females), three *T. bernacchii* individuals (1 male and 2 females) and two *G. acuticeps* individuals (1 male and 1 female) used for transcriptome and miRNA analyses were collected from Prize Bay, Antarctica (69°22′S, 76°22′E).

Thirty adult zebrafish individuals (15 males and 15 females) acclimated to hypoxia (DO = 1.0 ± 0.2 mg/L) or normoxia (DO = 6.5 ± 0.2 mg/L) for 2–3 weeks were used for miRNA analysis.

#### Cell lines

The H9C2 cardiomyocytes were cultured in DMEM medium with 10% fetal bovine serum (FBS) and maintained in an atmosphere of 5% CO2 at 37°C either under 21% normoxia or 1% hypoxia environment.

### Method details

#### Sample collection and biological parameter measurement

In order to evaluate the enlargement of icefish heart, we compared the heart-to-body mass ratio between the white-blooded *C*. *hamatus* and red-blooded notothenioids *T*. *bernacchii.* The two study species were collected from Ross Sea, Antarctica (74°55′S, 163°46′E) with the water depth of 130-160m. Biological data including body mass and length were measured. 150 mg/L Tricaine Methanesulphonate (MS-222) was used for the anesthetic, which was buffered with sodium bicarbonate to pH = 7 before immersing the fish. The fishes were left in the solution for at least 30 min following cessation of opercular movement to ensure death, heart tissues were immediately removed from the fish and heart weights were measured. Heart-to-body mass ratio of each fish was then acquired. Ten *C*. *hamatus* (5 males and 5 females) and ten *T*. *bernacchii* individuals (5 males and 5 females) were used for the measurement.

#### Paraffin sectioning and hematoxylin-eosin staining

The ventricle from *C. hamatus* and *T. bernacchii* hearts were dissected, respectively, fixed in a 10% buffered formalin solution, and embedded in paraffin. All of the paraffin sections are 5 μm thick, obtained from 10 formalin-fixed, paraffin-embedded archival fish cardiac tissue. Paraffin was removed from the section by immersing the slide in two xylene changes for 5 min each. Hydrate twice in 100% ethanol for 5 min, followed by 3 min with 95%, 90%, 80%, and 70% ethanol, respectively. Rinse in distilled water for 3 min, then rinse with PBS for 3 × 5 min. These dewaxed and rehydrated sections are treated with hematoxylin-eosin (HE) and examined under a Carl Zeiss microscope. In order to evaluate cardiomyocyte morphological difference between the red-blood and white-blooded fish, 3 different individuals for the two Antarctic fish species were used and 3 randomly chosen square areas (50 × 50μm2) per one individual were analyzed. Moreover, to compare the cardiomyocytes size between *C. hamatus* and *T. bernacchii*, cross-section sizes from randomly selected 50 cardiomyocytes were determined using Image J software. The statistical significance was determined using a two-tailed unpaired Student’s t test with *p* < 0.05.

#### Transcriptome sequencing

The three study species used for transcriptome analysis were lively collected from Prydz Bay, Antarctica (69°22′S, 76°22′E). The experiment procedures were in accordance with the Animal Care and Use Committee guidelines of Shanghai Ocean University (SHOU-DW-2012-002). 150 mg/L Tricaine Methanesulphonate (MS-222) was used for the anaesthesia. After fishes were anaesthetized for at least 30 min to ensure death, heart tissues were immediately removed from 3 *C. hamatus* (1 male and 2 females), 3 *T. bernacchii* (1 male and 2 females) and 2 *G. acuticeps* (1 male and 1 female) individuals and preserved immediately in −80°C freezer for further processing. The samples were subjected to RNA isolation using TRIZOL Reagent (Invitrogen, Carlsbad, CA). The quantity of total RNA was determined using a Qubit fluorimeter (Life Technologies). Six μg of total RNA from each sample were used to prepare the RNA-Seq library with TruSeq RNA Sample Prep Kit V2 (Illumina) following the manufacturer’s instructions. RNAseq libraries were quantified using an Agilent 2100 Bioanlyzer (Agilent Technologies). The libraries were then sequenced on an Illumina HiSeq 1500 platform (Illumina) with 100 bps paired-end reads. Around 10–20 million raw reads for each library were generated (CH_1: 12.6 M, CH_2: 11.2 M, CH_3: 18.0 M, GA_1: 16.4 M, GA_2: 18.4 M, TB_1: 9.3 M, TB_2: 13.6 M, TB_3: 21.9 M). The reproducibility of biological replicates was evaluated by the Pearson’s correlation coefficient, and the coefficient of determination (r2) for the samples ranged from 0.82 to 0.95 (Figure S6).

#### Sequence assembly and annotation

Clean reads with a Phred score ≥20 were kept for assembly. To ensure the same order and integrity of the paired-end reads, the custom Perl script used in the Manfred G. Grabherr et al. article is cited to filter double-ended fastq files. The cleaned reads that were paired were kept for the *de novo* assembly using the Trinity^76^ (version 2.4.0) software set with the default parameters.

The assembled unigenes were first aligned to the public National Center for Biotechnology Information (NCBI) non-redundant (nr) or SwissProt protein databases by BlastX with an E-value ≤1.0e−5. The unigene was assigned by the identity of the protein with the highest sequence similarity, which was used for functional annotation using the Blast2GO.^77^

#### Reads mapping and differential gene expression detection

The paired reads of each sample were mapped to the assembled unigenes with Bowtie.^78^ The expression abundance was estimated with RSEM^79^ implemented in the Trinity package using Fragments Per Kilobase of exon per Million fragments mapped (FPKM). To deduce the differential gene expression patterns for the three notothenioids, the predicted protein set (∼22,500 proteins) derived from *Dissostichus mawsoni,* a notothenioid species whose genome is fully sequenced, was used as the common ref.^80^ Differential expression detection was performed with the DESeq2 package (version: 1.2.9).^81^ Genes were deemed to be significantly differentially expressed with a adjusted *p*-value (FDR) ≤ 0.01 and an estimated absolute log2-fold change ≥1 after the Benjamini-Hochberg correction. Only the genes presenting changes in the same direction in *C. hamatus*/*G. acuticeps* and *C. hamatus*/*T. bernacchii* comparisons were considered differentially expressed genes (DEGs) between the icefish and the red-blooded fish. GO enrichment analysis was conducted with the DEGs using R based on a hypergeometric distribution. Significantly enriched GO terms were identified based on the corrected *p*-value (*p* < 0.05).

#### Validation of gene expression by quantitative reverse transcription real-time PCR (qRT-PCR)

Two micrograms of RNA from *C. hamatus* and *T. bernacchii* heart tissues were reverse transcribed to cDNA using an RT-PCR kit (TaKaRa). qPCR was performed using SYBR Green Master Mix following the manufacturer’s protocol (Roche). *β*-Actin was used as an internal control. The primers used are listed in Table S17. RT-PCR was performed as follows: 95°C for 3 min, 35 cycles at 95°C for 30 s, 54°C–64°C for 30 s and 72°C for 20 s; and final extension at 72°C for 3 min. All of the reactions were performed with three biological replicates, and each biological sample was assayed three times. The average value was calculated from biological and technical repeats. The relative expression levels of the genes were calculated using the 2−ΔΔCT method. The statistical significance was determined using a two-tailed unpaired Student’s t test with *p* < 0.05.

#### Western blot analysis

Proteins from heart tissues of several *C. hamatus* and *T. bernacchii* individuals were extracted and equivalent amounts of the proteins from the preparations were separated on SDS-PAGE gels and, then, transferred onto a PVDF membrane (Millipore). The primary antibodies (Nkx2.5 (Santa Cruz Bio, sc-376565); Tbx 2 (OriGene, TA344550); Gata4 (GeneTex, GTX113194); Bmp2 (HuaAn Bio, ER80602); Bmp4 (GeneTex, GTX128348); Bmp7 (Sigma, QC49491); Smad1/5/9 (Abcam, ab66737); Mef2A (GeneTex, GTX50398), Ccna2 (LSBio, LS-C31034), and β-actin antibody (HuaAn Bio, M1210-2) were diluted to appropriate concentrations with 1X PBST. Primary antibodies were selected from the above commercial sources based on conservation of the epitope sequences in the target proteins of the Antarctic notothenioids. We examined the immunogen sequence of each antibody in the target proteins of *C. hamatus* and *T. bernacchii*. Color detection was performed with an enhanced chemiluminescence (ECL) reagent kit (Millipore). Western blot analyses were repeated at least three times in different individuals of each species. For each antibody, we performed western bolt comparisons between the *C. hamatus* and *T. bernacchii* samples in one gel. Different gels were run for biological replicates with two species loaded within the same gel. To minimize the inter-gel differences, we applied the same amount of extracted proteins and followed exactly the same procedures to conduct western blot and color development. Similar intensities for the target bands were produced between the gels for each antibody. The relative expression of each protein was quantified by densitometry using Image J (version 1.8.0). Expression index is calculated based on the ratio of band intensity of the target protein to that of *β*-actin. The statistical significance was determined using Student’s t test with *p* < 0.05.

#### Small RNA library preparation and sequencing for three Antarctic fishes

Total RNAs from the heart tissues of three individuals of *C. hamatus*, *T. bernacchii* and *G*. *acuticeps* were extracted using the miRNeasy Mini kit (Qiagen). Equal amounts of total RNA from three individuals of each species were mixed to obtain three pooled samples. The three small RNA libraries were constructed with the same conditions, and sequenced on an Illumina HiSeq2000 platform (Personalbiol, Shanghai, China). The raw reads were processed by trimming the poor-quality reads. Other RNAs (rRNA, tRNA, snRNA and snoRNA) were removed by blasting against the GenBank database (http://blast.ncbi.nlm.nih.gov) and the Rfam database (http://sanger.ac.uk/software/Rfam). The number of Clean Reads (Total Reads) with sequence lengths above 15 nt and below 30 nt were counted and de-duplicated for identical sequences in a single sample. The clean reads were searched against the known precursor/mature miRNAs in miRbase 22.0. We used the fish miRNAs in miRNAbase 22.0 for searching. Conserved miRNAs were screened from them, and the screening criteria were either exact match or mismatches did not exceed 2. And multiple sequence alignment of the interested miRNA sequences of icefish with those of the other vertebrates were conducted to evaluate the icefish sequences. The miRNA expression level was homogenized based on the total sequencing reads of each library to obtain the homogenization value Counts per million (CPM). The total number of clean reads was used as the denominator when calculating the CPM. Differentially expressed miRNAs (DEMs) were analyzed based on the fold difference in expression level (|log2 fold change|≥1) and the significance of the expression difference (*p*-value <0.05) using DESeq (Version 1.18.0). To compare the expression of miRNAs between *C. hamatus*/*G*. *acuticeps,* and *C. hamatus/T. bernacchii*, the data were evaluated on a log2-ratio plot. MiRNAs were deemed to be significantly differentially expressed with an estimated absolute log2-fold change≥1 and with≥15 CPM in at least one sample (If *C. hamatus* has lower expression in one miRNA, both *G. acuticeps* and *T. bernacchii* should have≥15 CPM). Only the miRNAs showing changes in the same direction in *C. hamatus*/*G. acuticeps* and *C. hamatus*/*T. bernacchii* comparisons were considered differentially expressed between the icefish and the red-blooded fish.

#### Quantitative miRNA real-time PCR assay

Small RNAs (<200 nt) from total RNA preparations of *C. hamatus* and *T. bernacchii* heart tissues were isolated using the mirVanaTM miRNA Isolation kit (Ambion) and then polyadenylated using poly(A) polymerase (Ambion). Same amount of small RNAs (<200 nt) *C. hamatus* and *T. bernacchii* heart tissues were isolated, polyadenylated and reverse transcribed. The cDNA was amplified by real-time PCR using the FastStart Universal SYBR Green Master (Roche). U6RNA was used as an internal control. The primers used are listed in Table S18. All of the reactions were performed with three biological replicates, and each biological sample was assayed three times. The average value was calculated from biological and technical repeats. The relative expression levels of the miRNAs were calculated using the 2−ΔΔCT method. The statistical significance was determined using a two-tailed unpaired Student’s t test with *p* < 0.05.

#### MiRNA target prediction

The 3′ untranslated regions (3′UTRs) of eight cardiac development genes (*bmp2, mef2a, mef2c, gata4, gata6, tbx2, tbx20 and hand2*) were cloned from the *C. hamatus* heart RNAs. The 3′ UTR sequences of the eight heart development genes are detailed in Table S8. The potential target genes for miR-458-3p and miR-144-5p were predicted using the miRanda v3.3a Target Scanning algorithm.^82^

#### MiRNA-458-3p antagomir and miR-144-5p antagomir microinjection and heart size measurement

The GMR-miRTM miRNA single-stranded antagomir for miR-458-3p (5′CAGUACCAUUUAAAGAGCUAU3′) and miR-144-5p (5′ ACUUACAGUAUAAGAUGAUAUCCU 3′) were synthesized (Shanghai Gene Pharma Co. Ltd.). A single-stranded RNA 5′UUCUCCGAACGUGUCACGUTT3′ was also synthesized as a negative control (NC miRNA). Tg *Danio rerio* (myl7: EGFP) (China Zebrafish Resource Center, CZRC) were used for the microinjection experiment. Fertilized 1- to 2-cell embryos were microinjected with 1 nL of the synthetic miRNA (50 μM). Images were taken at 200X magnification with a Carl Zeiss microscope at 72 h post-fertilization. The heart including atrium and ventricle was outlined by a smooth curve provided by ZEISS ZEN 2012 Image Software, and the heart sizes were subsequently calculated based on the drawn heart shape and compared among the different groups of antagomir injections. The quantitative data for heart measurement for WT, NC and miR-458-3p (miR-144-5P) were derived from 15 independent embryos. The statistical significance was determined using a two-tailed paired Student’s t test with *p* < 0.05.

#### Cell transfection assays of miRNA-458 antagomir and miR-144-5p antagomir

The H9C2 cardiomyocytes were cultured in DMEM medium with 10% fetal bovine serum (FBS) and maintained in an atmosphere of 5% CO2 at 37°C either under 21% normoxia or 1% hypoxia environment. The miR-458-3p antagomir and miR-144-5p antagomir was transfected into cells with HiPerFect Transfection Reagent (QIAGEN), respectively. The final concentration of miRNA antagomir and NC miRNA for transfection was 20nM. The transfected H9C2 cardiomyocytes were cultured in a 6-well plate for 24 h, and then the cells were collected for counting the number by Nexcelom Cellometer (USA). The cell numbers were compared with those from WT (wild type) and NC transfected cells. The statistical significance was determined using a two-tailed paired Student’s t test with *p* < 0.001.

#### Microinjection of antagomir and mimics of miR-458-3p and miR-144-5p and cardiomyocytes proliferations detection

All handling of fish was carried out in accordance with the guidelines on the care and use of animals for scientific purposes set up by the Institutional Animal Care and Use Committee (IACUC) of Shanghai Ocean University (IACUC SHOU-DW-20171022). The ventricular resection experimental procedure followed Poss et al.’s the approach.^83^ Eighteen 1-to 2-year-old adult wild-type zebrafish (∼0.3g) were fasted for 24 h and then put into 150 mg/L Tricaine Methanesulphonate (MS-222) solution. The anaesthetic fish individuals were placed ventral side up into a moist, slotted sponge. Iridectomy scissors were used to make a small incision that penetrated the skin and pericardial sac. The ventricle was exposed by gentle abdominal pressure, and 15% of the ventricle at the apex was removed by scissors. The 15% ventricular resection surgical procedures were followed the approach of Poss et al.*.*^83^

The apex cordis excisional zebrafish individuals were microinjected by 1.5ul EDPC water, NC miRNA (1.5 μl, 0.05nmol/μl), miR-458-3p mimics (1.5 μl, 0.05nmol/μl), miR-458-3p antagomir (1.5 μl, 0.05nmol/μl), miR-144-5p mimics (1.5ul, 0.05nmol/μl) and miR-144-5p antagomir (1.5ul, 0.05nmol/μl), respectively. For each microinjection, 2.5ul Edu (5-Ethynyl-2′-deoxyuridine) (Guangzhou Shuopu Biotechnology Co., Ltd.) was together microinjected into zebrafish abdomen. All of the microinjections for each miRNA were performed with three biological replicates. 72 h after microinjection, fishes were anaesthetized, and hearts were removed and fixed in 4% paraformaldehyde, and embedded in paraffin. After deparaffinization, Apollo fluorescence staining and DNA staining were performed by using the Cell-Light EdU Apollo567 *In Vitro* Kit (Guangzhou Ribo Biotechnology Co., Ltd.). After staining, we used the confocal laser scanning microscope (DMi8TCSSP8, Leica, Germany) to observe and photograph to observe the cardiomyocytes proliferation (red fluorescent dots indicate proliferation signals).

#### Verification of the expression level of miR-458-3p and miR-144-5p in the zebrafish heart by quantitative miRNA real-time PCR assay

MiR-458-3p antagomir, miR-144-5p antagomir and NC were synthesized and micro-injected into fertilized 1- to 2-cell zebrafish embryos. The embryo hearts were cut at 72 h post-fertilization for WT, NC micro-injection and miR-458-3p antagmir micro-injection (miR-144-5p antagmir micro-injection) groups. Small RNAs from total RNA preparations of the zebrafish heart tissues were isolated and the expression of miR-458-3p (miR-144-5p) was compared between the WT, NC and miR-458-3p antagmir micro-injection (miR-144-5p antagmir micro-injection) groups using ‘quantitative miRNA real-time PCR assay’ protocols described above. The primers used are listed in Table S18. At least three biological replicates were performed for each measurement. The statistical significance was determined using a two-tailed paired Student’s t test with *p* < 0.05.

#### Dual luciferase and EGFP reporter assays

The 3′UTRs of *C. hamatus bmp2* gene were amplified by PCR via reverse transcription from the heart total RNA samples. *bmp2* 3′UTR luciferase reporter plasmids were constructed using the pMIR-REPORT Luciferase vector.^84^ The pMIR-*bmp2*-3′UTR was constructed using the following primers: forward, 5′-CGAGCTCCGAGGGAATAGGAGGAAGAC-3′, and reverse, 5′-GCGTCGACGTTTGCTTTGTTTATTAGAGGTA-3’.

In addition, the EGFP reporter plasmids were constructed using the pTOL2-bactin-2A-EGFP vector.^76^ The pEGFP-*bmp2*-3′UTR was obtained using the following primers: forward, 5′-GCGGCCGCCGAGGGAATAGGAGGAAGAC-3’; reverse, 5′- CAATTGGTTTGCTTTGTTTATTAGAGGTA-3’. The 3′UTR of *C. hamatus bmp2* gene was cloned into the TOL2 vector (Life Technologies) (Figure S2). All the recombinant plasmids were checked by DNA sequencing and restriction enzyme digestion.

293T cells were co-transfected with the recombinant plasmids pMIR-*bmp2*-3′UTR and miR-458-3p mimics (miR-144-5P mimics) or NC miRNA (Negative Control miRNA, 5′UUGUACUACACAAAAGUACUG 3′) for dual luciferase assays. The luciferase activity was measured using a Dual-Luciferase Reporter Assay System (Promega, USA), and the intensity of green fluorescence was examined with a fluorescent microscope (CarlZeiss, Germany).

H9C2 cells were cotransfected with 1.2ug TOL2-GFP-*bmp2* 3′UTR vector, 40 μmol miR-458-3p mimics (miR-144-5p mimics) or NC miRNA (Negative Control miRNA, 5′UUGUACUACACAAAAGUACUG 3′) with 0.01 μg Attractene Transfection (QIAGEN). The intensity of green fluorescence was examined using a fluorescent microscope (Carl Zeiss, Germany) at 48 h post transfection. To check the inhibitory effect of the *bmp2* 3′UTR by the miRNAs, wild-type TU stocks of *Danio rerio* were used for the EGFP reporter assays. *In vivo*, fertilized 1- to 2-cell embryos were co-microinjected with TOL2-GFP-*bmp2* 3′UTR recombinant plasmid, miR-458-3p mimics (miR-144-5p mimics) or NC. The embryos were collected for examining GFP protein expression (GFP primary antibody, GT859, GeneTex) by western blot.

#### Small RNA library preparation and sequencing for hypoxia acclimated and normoxia zebrafish heart

Thirty adult zebrafish individuals (15 males and 15 females) were acclimated to hypoxia (DO = 1.0 ± 0.2 mg/L) or normoxia (DO = 6.5 ± 0.2 mg/L) for 2–3 weeks. We followed the NIH guidelines for zebrafish euthanasia (https://oacu.oir.nih.gov/sites/default/files/uploads/arac-guidelines/zebrafish.pdf). 150 mg/L Tricaine Methanesulphonate (MS-222) was used for the anaesthesia. Fish were be left in the ice water for at least 30 min after cessation of all movement to ensure death and hearts were removed. Total RNAs from the heart tissues of thirty individuals of DO = 1.0 ± 0.2 mg/L hypoxia acclimated zebrafish and DO = 6.5 ± 0.2 mg/L normoxia acclimated zebrafish were extracted using the miRNeasy Mini kit (Qiagen). The procedure for small RNA sequencing and analysis of the hearts of hypoxia-acclimated and normoxia zebrafish were similar to the small RNA sequencing and analysis for three Antarctic fishes described previously (See the Section “small RNA library preparation and sequencing for three Antarctic fishes”). To compare the expression of miRNAs between Hypoxia/Normoxia, the data were evaluated on a log2-ratio plot. MiRNAs were deemed to be significantly differentially expressed with an estimated absolute log2-fold change≥1 and with≥15 CPM in at least one sample.

#### Expression of miR-458-3p, miR-144-5p and *bmp2* in hypoxia acclimated zebrafish heart

One hundred and twenty adult zebrafish individuals divided into two experimental groups (60 individuals for each group, including 30 males and 30 females) were acclimated to hypoxia (DO = 1.0 ± 0.2 mg/L) or normoxia for 2–3 weeks. Fishes were anaesthetized and hearts were removed for isolation of total RNA and protein. Small RNAs from total RNA preparations of hypoxia (DO = 1.0 ± 0.2 mg/L) or normoxia zebrafish heart tissues were isolated and the expression of miR-458-3p and miR-144-5p was compared between hypoxia (DO = 1.0 ± 0.2 mg/L) or normoxia zebrafish heart using ‘quantitative miRNA real-time PCR assay’ protocols described above. Expression of the genes of interest was measured using the RT-PCR protocols described above. Heart tissues from hypoxia (DO = 1.0 ± 0.2 mg/L) or normoxia (DO = 6.5 ± 0.2 mg/L) fishes were also used for detection of *bmp2* protein expression. At least three biological replicates were performed for each measurement.

#### Expression of miR-458-3p, miR-144-5p and *bmp2* in hypoxic Antarctic fish heart

Six red-blooded notothenioids *T. bernacchii* individuals were collected from Ross Sea, Antarctica (74°55′S, 163°46′E) in January 2018. All the six *T. bernacchii* individuals were cultivated with seawater (DO = 8.9 ± 0.2 mg/L) in an open breeding tank placed on the deck for at least 24 h. Low oxygen seawater environment (DO = 6.0 ± 0.2 mg/L) was created by constantly bubbling nitrogen into the water. Three *T. bernacchii* individuals were randomly selected out and exposed to hypoxia environment (DO = 6.0 ± 0.2 mg/L) for 48 h. The DO level (DOL) was measured with a portable DO meter (YSI Pro20). Three individuals under normoxia environment (DO = 8.9 ± 0.2 mg/L) were set as control group. 150 mg/L Tricaine Methanesulphonate (MS-222) was used for the anaesthesia. After fishes were anaesthetized the on ice for at least 30 min to ensure death, and hearts were removed for isolation of total RNA and protein. Small RNAs from total RNA preparations of hypoxic or normoxia conditioned *T. bernacchii* heart tissues were isolated and the expression of miR-458-3p and miR-144-5p was compared between hypoxic or normoxia *T. bernacchii* heart using ‘quantitative miRNA real-time PCR assay’ protocols described above. Heart tissues from hypoxic and normoxia *T. bernacchii* were also used for detection of *bmp2* protein expressions. At least three biological replicates were performed for each measurement.

### Quantification and statistical analysis

#### Statistical analysis

Data were analyzed using GraphPad Prism 5.0 software. All data are expressed as mean ± standard error (SEM). Differences between two groups were compared using an unpaired Student’s t-test. Differences between three or more groups were analyzed using one-way ANOVA. All tests were two-tailed, and treatments with significant differences (*p* < 0.05, *p* < 0.01, *p* < 0.001, Student’s t test) are indicated by “∗”, “∗∗” and “∗∗∗” respectively. N.S. is an abbreviation for non-significant. Error bars indicate ±1 SEM.

## Supplemental information

Document S1. Figures S1‒S6 and Tables S1–S3, S5–S7, S9–S12 and S14–S18

Table S4. Significantly up or down-regulated genes in the heart of the Chionodraco hamatus compared with other two red-blooded Antarctic fish, related to Figure 2

Table S8. 3′ UTR of eight cardiac development genes, related to Table 1

Table S13. Differentially expressed miRNAs between the hypoxic and normoxic zebrafish heart
