# Supplementary material for: Identification of two miRNAs regulating cardiomyocyte proliferation in an Antarctic icefish
Source: iScience. 2024 May 27;27(6):110128. doi: 10.1016/j.isci.2024.110128 (PMC11209021; doi:10.1016/j.isci.2024.110128)
Supplement: Document S1. Figures S1‒S6 and Tables S1–S3, S5–S7, S9–S12 and S14–S18 [file mmc1.pdf]

iScience, Volume *27*

Supplemental information

Identification of two miRNAs regulating cardiomyocyte proliferation in an Antarctic icefish

Qianghua Xu, Ruonan Jia, Fei Yang, Peng Hu, Xue Li, Saiya Ge, Shouwen Jiang, Jiulin Chan, Wanying Zhai, and Liangbiao Chen

Supplementary Figures

Supplementary Fig.1 Genetic evolutionary tree of three Antarctic fish species used in this study,related to Figure 1.

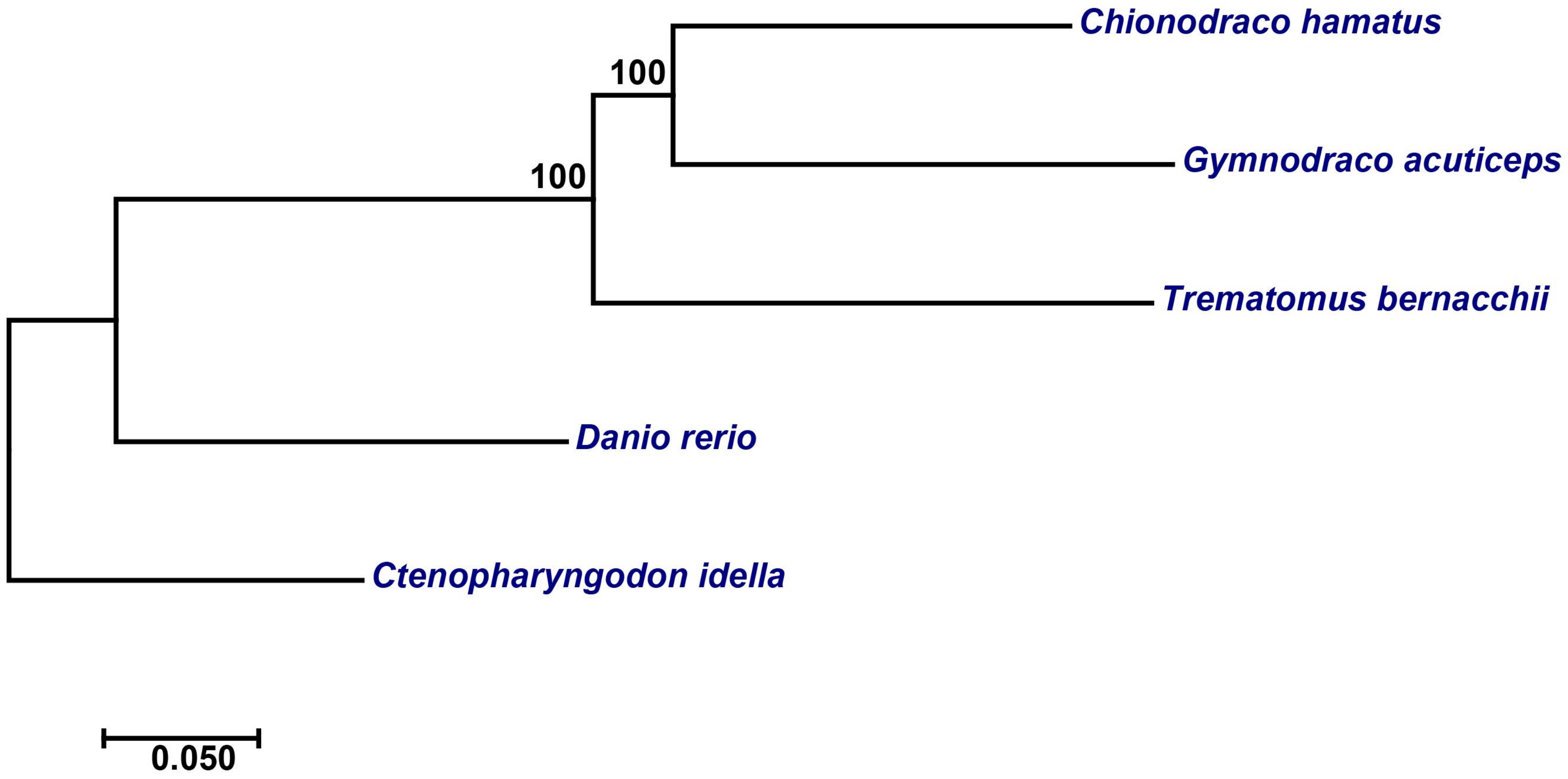

Mitochondrial genome sequences of three Antarctic fishes and two other fishes were downloaded from the NCBI database to produce genetic evolutionary tree.(**Accession number** *Chionodraco hamatus* : KU341409.1; *Gymnodraco acuticeps* : NC 057669.1; *Trematomus bernacchii* : NC 053364.1; *Danio rerio*: NC 002333.2 ; *Ctenopharyngodon ideiia* : NC 010288.1). The evolutionary history was inferred using the Neighbor-Joining method. The optimal tree with the sum of branch length = 1.01440064 is shown. The percentage of replicate trees in which the associated taxa clustered together in the bootstrap test (1000 replicates) are shown next to the branches. The tree is drawn to scale, with branch lengths in the same units as those of the evolutionary distances used to infer the phylogenetic tree. The evolutionary distances were computed using the p-distance method and are in the units of the number of amino acid differences per site. The analysis involved 5 amino acid sequences. The coding data was translated assuming a Standard genetic code table. All ambiguous positions were removed for each sequence pair. There were a total of 7495 positions in the final dataset. Evolutionary analyses were conducted in MEGA7.

Supplementary Fig.2 Schematic drawing of the pTOL2-EGFP recombinant plasmid, related to Figure 6.

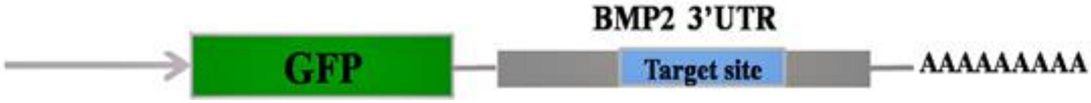

The EGFP reporter plasmids were constructed using the pTOL2-bactin-2A-EGFP vector. The pEGFP-bmp2-3'UTR was obtained using the following primers:
forward, 5'-GCGGCCGCCGAGGGAATAGGAGGAAGAC-3';
reverse, 5'- CAATTGGTTTGCTTTGTTTATTAGAGGTA-3'.

The 3'UTR of *C. hamatus bmp2* gene was cloned into the TOL2 vector (Life Technologies). All the recombinant plasmids were checked by DNA sequencing and restriction enzyme digestion.

H9C2 cells were co-transfected with TOL2-GFP-bmp2 3'UTR vector, miR-458-3p mimics (miR-144-5p mimics) or NC miRNA with Attractene Transfection (QIAGEN). TOL2-GFP-bmp2 3'UTR recombinant plasmid, miR-458-3p mimics (miR-144-5p mimics) or NC were also co-microinjected into 1- to 2-cell embryos.

Supplementary Fig.3 The expressing level of miR-458-3p and miR-144-5p between the hypoxic and nomorxic zebrafish heart, related to Figure 7.

miRNA Name	Hypoxic Heart CPM	Normoxic Heart CPM	Log2(Hypoxic/Normorxic)
dre-miR-458-3p	478	1934	-2.621
dre-miR-144-5p	2091	40644	-4.885

CPM indicates the Counts per million.

The two miRNAs, miR-458-3p and miR-144-5p were downregulated as seen in the hypoxic challenged zebrafish heart.

Supplementary Fig.4 volcano plots for the RNA-seq and small RNA-seq data, related to Figure 2 and Figure 7.

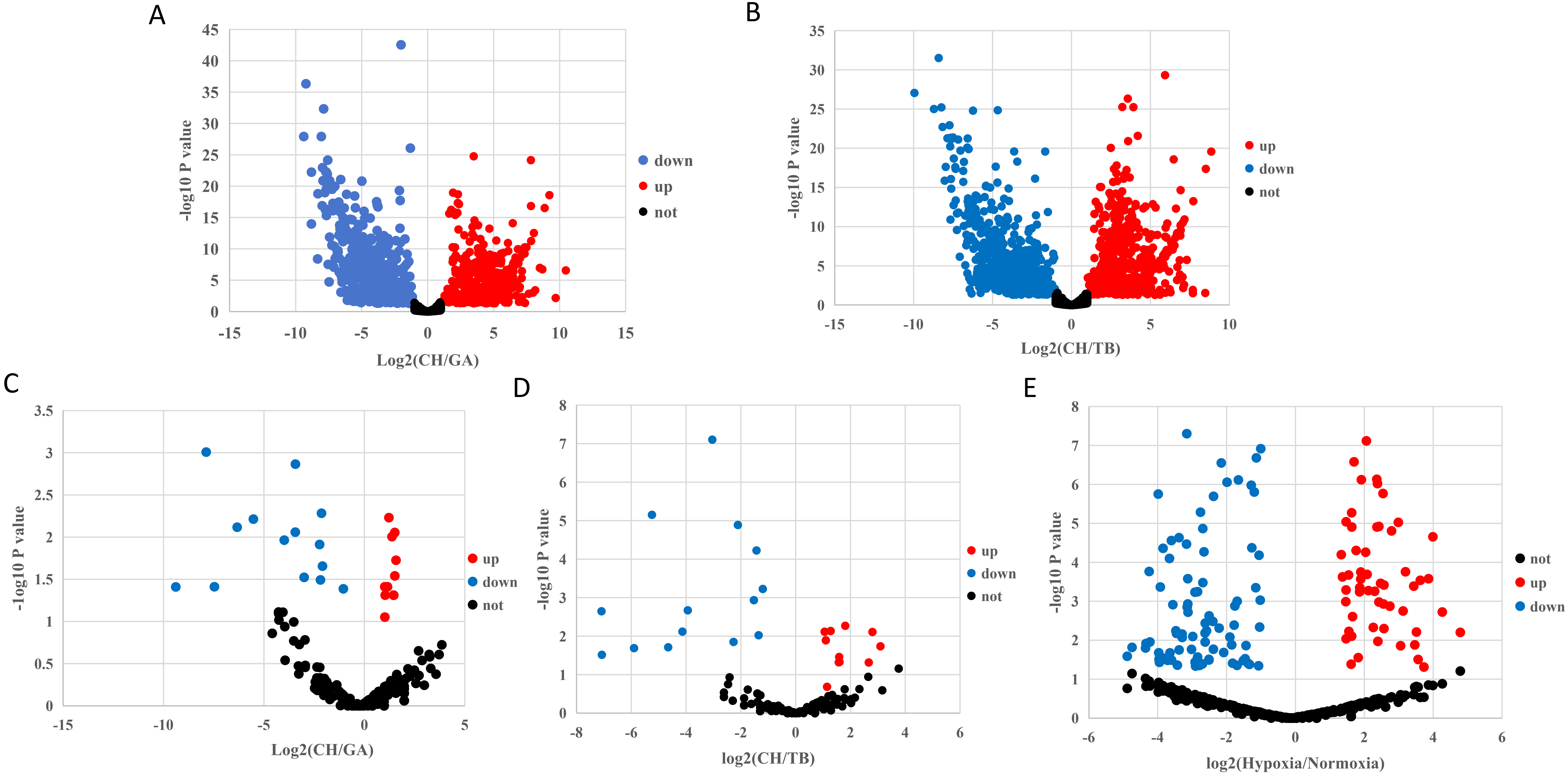

Horizontal coordinates represent the multiplicity of changes in gene expression in different samples, vertical coordinates represent the statistical significance of changes in gene expression, and scattered dots in the graph represent individual genes or miRNAs, gray dots represent genes or miRNAs with no significant differences, red dots represent significantly up-regulated differential genes or miRNAs, and blue dots represent significantly down-regulated differential genes or miRNAs.(A) Comparison of CH and GA differential genes under RNA-seq sequencing; (B) Comparison of CH and TB differential genes under RNA-seq sequencing; (C) Comparison of CH and GA differential mirrors under small RNA-seq sequencing; (D) Comparison of CH and TB differential mirrors under small RNA-seq sequencing; (E) Comparison of zebrafish small RNA-seq sequenced differential mirna under hypoxic and normoxic conditions.

Supplementary Fig.5 Sequence comparisons of miR-458-3p , miR-144-5p and *bmp2* binding sites to the two miRNAs, related to Figure 6.

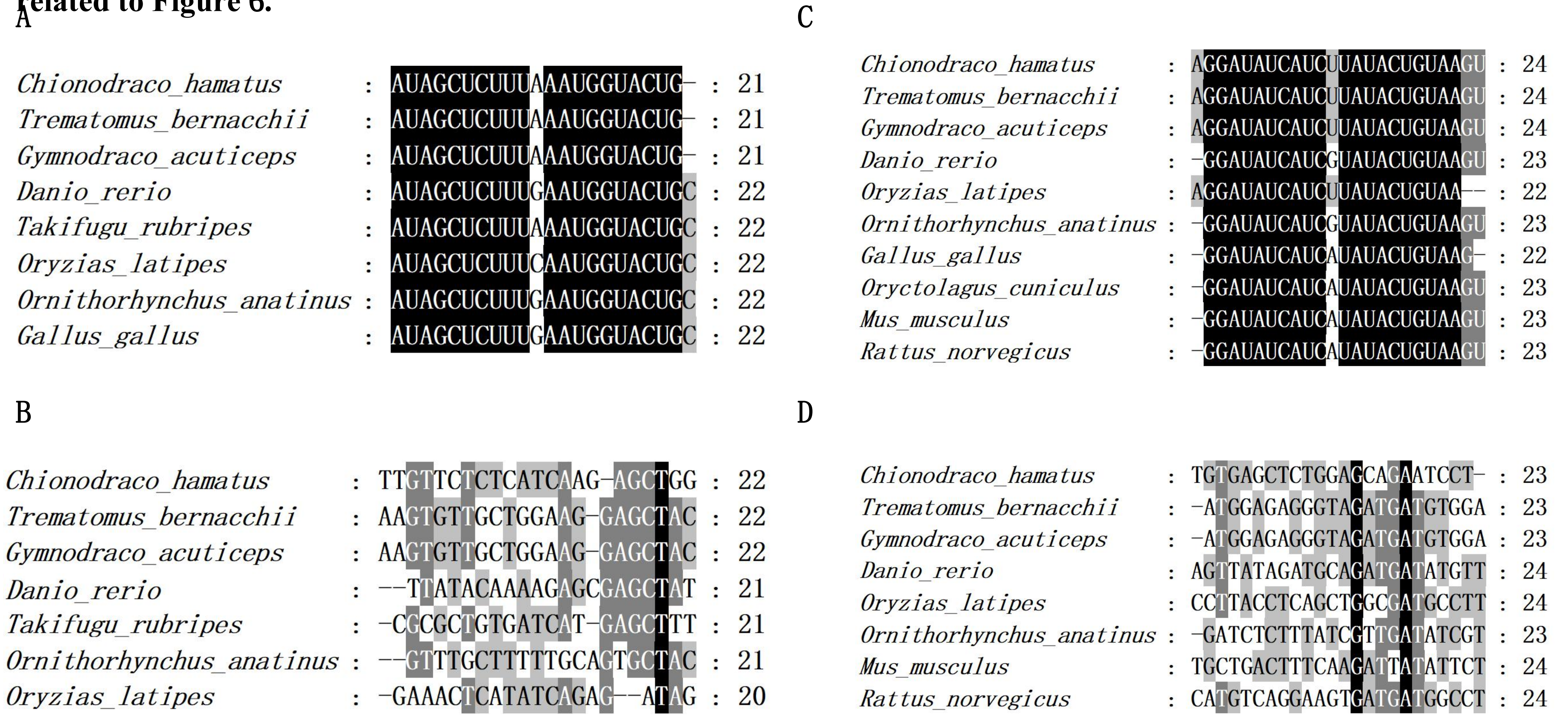

The miRNA mature sequences were downloaded using miRbase database, and the *bmp2* 3'UTR sequences were downloaded using NCBI database. target sequence prediction was performed using miRanda v3.3a software, and sequence comparison was performed and plotted using MEGA and GeneDoc software, with black shading representing a sequence conserved percentage of 100, gray representing a conserved percentage of 70, and light gray representing a conserved percentage of 40.

(A) **miR-458-3p** (MI0002179 *Danio rerio*; MI0003252 *Takifugu rubripes*; MI0019477 *Oryzias latipes*; MI0006789 *Ornithorhynchus anatinus*; MI0007567 *Gallus gallus*) .

(B) ***bmp2* binding sites to miR-458-3p** (NM_131360.2 *Danio rerio*; XM_003971524.3 *Takifugu rubripes*; XM_007672096.4 *Ornithorhynchus anatinus*; NM_001104908.1 *Oryzias latipes*) .

(C) **miR-144-5p** (MI0002009 *Danio rerio*; MI0019501 *Oryzias latipes*; MI0006723 *Ornithorhynchus anatinus*; MI0004996 *Gallus gallus*; MI0039302 *Oryctolagus cuniculus*; MI0000168 *Mus musculus*; MI0000917 *Rattus norvegicus*).

(D) ***bmp2* binding sites to miR-144-5p** (NM_131360.2 *Danio rerio*; NM_001104908.1 *Oryzias latipes*; XM_007672096.4 *Ornithorhynchus anatinus*; NM_007553.3 *Mus musculus*; NM_017178.2 *Rattus norvegicus*) .

Supplementary Fig.6 Comparisons between the biological replication data sets of the *C. hamatus* (CH_1, CH_2 and CH_3) *G. acuticeps* (GA_1 and GA_2) and *T. bernacchii* (TB_1, TB_2 and TB_3),related to Figure 2 .

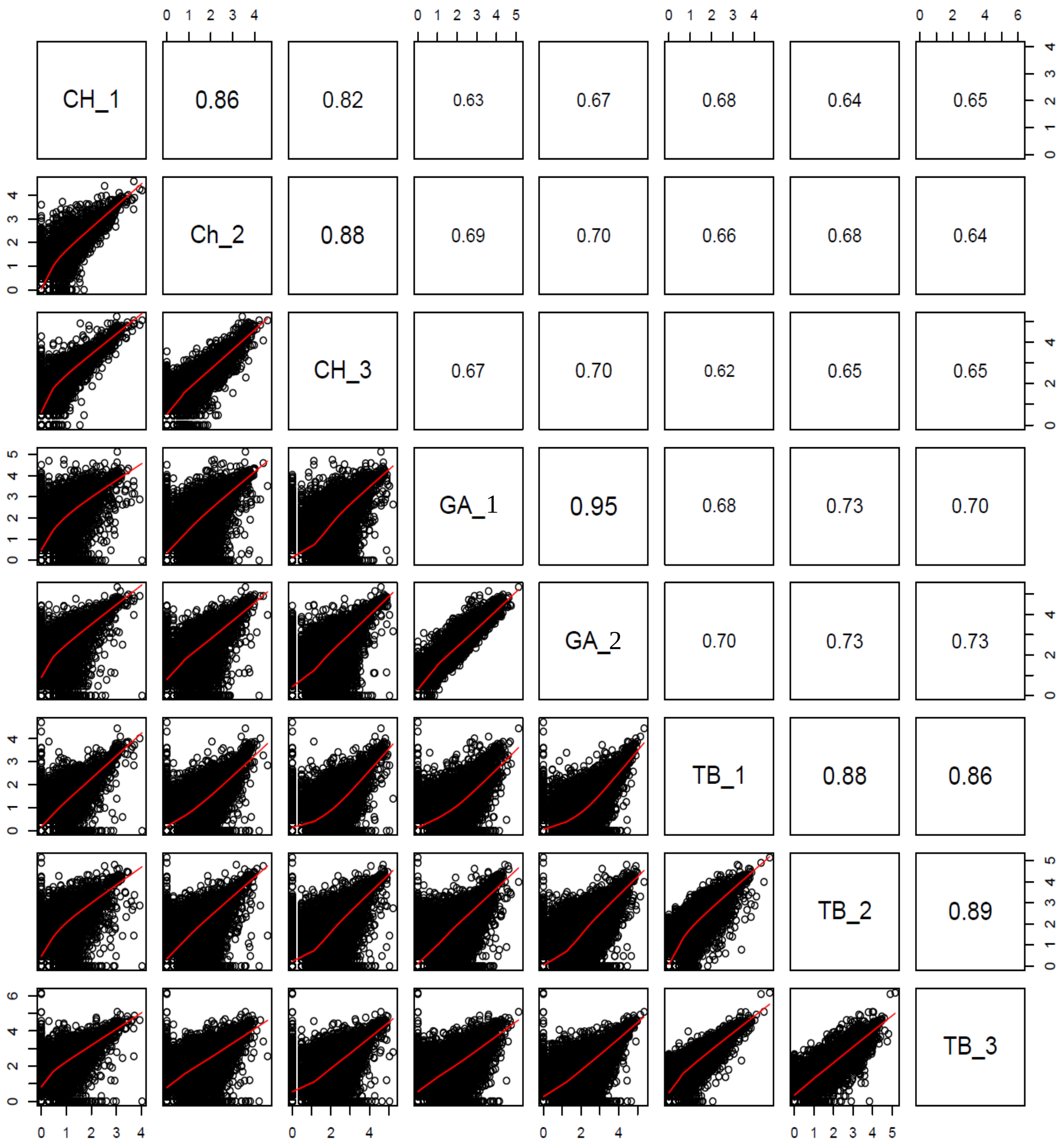

The mapped reads of 12,774 unigenes were shown in scatterplot. Axis represents the biological replica. The Pearson's correlation coefficients (r) between the two replicates were calculated and the coefficients of determination (r^2) were shown in the plots.

Supplementary Table 1. Body mass, length, and heart-to-body mass ratio of *T. bernacchii* and *C. hamatus*, related to Figure 1.

T. bernacchii					*C. hamatus*				
	Length(mm)	Body mass (g)	Heart Weight (g)	Heart/Body (%)		Length(mm)	Body mass (g)	Heart Weight (g)	Heart/Body (%)
1	216	260	0.231	0.082	1	289	305	1.236	0.405
2	215	216	0.201	0.093	2	344	475	1.698	0.357
3	198	152	0.129	0.085	3	367	360	1.431	0.397
4	244	167	0.162	0.097	4	354	235	1.039	0.442
5	234	268	0.252	0.094	5	355	345	1.267	0.367
6	300	431	0.375	0.087	6	371	435	1.433	0.329
7	192	105	0.109	0.104	7	305	450	1.773	0.394
8	210	161	0.156	0.098	8	345	340	1.308	0.385
9	248	212	0.216	0.102	9	317	345	1.298	0.376
10	205	135	0.152	0.113	10	368	495	1.988	0.402
Average	226	211	0.198	0.0955	Average	342	378	1.450	0.3854
SD	32	93.7	0.077	0.0094	SD	38.4	82.5	0.288	0.0307

Note: The pictures of *T. bernacchii* and C. hamatus's heart shown on Figure 1 were from Number 1 fish samples of the two fish species.

Supplementary Table 2.Cardiomyocyte measurement of *T. bernacchii* and *C. hamatus*, related to Figure 1

	TB area(μm^2)	CH area(μm^2)		TB area(μm^2)	CH area(μm^2)
1	163	155	26	159	160
2	169	157	27	169	159
3	169	151	28	171	156
4	170	155	29	167	162
5	160	155	30	161	159
6	172	157	31	173	157
7	170	149	32	167	147
8	159	167	33	163	149
9	168	163	34	169	145
10	169	171	35	171	156
11	163	161	36	167	159
12	162	165	37	162	165
13	168	166	38	173	154
14	162	163	39	169	150
15	165	153	40	159	154
16	167	172	41	161	156
17	148	160	42	168	148
18	157	166	43	151	150
19	163	156	44	169	152
20	157	167	45	167	162
21	159	161	46	171	161
22	159	167	47	161	159
23	165	167	48	172	152
24	151	167	49	157	151
25	155	160	50	171	146
Average		*T. Bernacchii* 164	*C. hamatus* 158		
SD		*T. Bernacchii* 6	*C. hamatus* 7		
P-value		0.977			

Supplementary Table 3. Sample sequence statistics, related to Figure 2 and Figure 7.

RNA Sequencing data	raw reads	clean reads
CH1	12590361	12005341
CH2	11217314	10954620
CH3	17979383	15030812
GA1	16376945	15996257
GA2	18393272	17921979
TB1	9332071	9071250
TB2	13645727	13207134
TB3	21901606	18135557
**Small RNA Sequencing data**	**raw reads**	**clean reads**
Normoxia zebrafish heart	8177709	7931491
Hypoxia zebrafish heart	8587757	6351340
CH heart	11082426	11082304
GA heart	17248458	17247819
TB heart	15126182	15125996

Supplementary Table 5. Gene expression profiles of the transcriptional factors involved in heart development, related to Figure 3.

Protein ID	Protein Name	*C.hamatus/G.acuticeps*		*C.hamatus/T.bernacchii*		Description
		Log2(CH/GA)	*P* (CH/GA)	Log2(CH/TB)	*P* (CH/TB)	
P12643	BMP2_HUMAN	3.086	0.024050012	1.453	0.311216253	Bone morphogenetic protein 2
P12644	BMP4_HUMAN	0.090	0.9628101	1.640	0.166922163	Bone morphogenetic protein 4
P23359	BMP7_MOUSE	5.957	NA	6.359	NA	Bone morphogenetic protein 7
Q9I8V2	SMAD1_DANRE	4.397	1.05132E-06	2.128	0.015410394	Mothers against decapentaplegic homolog 1
Q9I9P9	SMAD2_DANRE	8.233	NA	5.495	NA	Mothers against decapentaplegic homolog 2
Q13485	SMAD4_HUMAN	0.257	0.852335163	2.843	0.000187749	Mothers against decapentaplegic homolog 4
O35182	SMAD6_MOUSE	1.193	0.560064642	1.909	0.255287099	Mothers against decapentaplegic homolog 6
O15198	SMAD9_HUMAN	4.626	NA	6.131	NA	Mothers against decapentaplegic homolog 9
A2VDZ3	MEF2A_BOVIN	7.141	5.89558E-18	1.033	0.252226477	Myocyte-specific enhancer factor 2A
Q02080	MEF2B_HUMAN	2.852	0.047313998	1.348	0.370563552	Myocyte-specific enhancer factor 2B
A4UTP7	MEF2C_PIG	5.774	1.02109E-10	2.995	0.000366005	Myocyte-specific enhancer factor 2C
Q14814	MEF2D_HUMAN	1.031	0.617327382	1.075	0.554407134	Myocyte-specific enhancer factor 2D
P11831	SRF_HUMAN	-0.698	0.627751539	-1.752	0.08931024	Serum response factor
P42583	NKX25_XENLA	-0.879	0.606738511	-0.513	0.750140145	Homeobox protein Nkx-2.5
Q7ZTU9	TBX2_DANRE	1.784	0.101056236	1.660	0.08931024	T-box transcription factor TBX2b
Q9PWE8	TBX5_CHICK	0.524	0.82744526	0.428	0.847371149	T-box transcription factor TBX5
Q9I9K7	TBX20_DANRE	-1.218	0.285815899	3.904	1.36191E-05	T-box transcription factor TBX20

Q91677	GATA4_XENLA	0.165	0.851527501	0.053	0.949883798	Transcription factor GATA-4
Q91678	GAT6A_XENLA	-0.657	0.429317456	-1.288	0.032503404	GATA-binding factor 6-A
Q90691	HAND1_CHICK	-4.376	1.0366E-08	-4.429	1.99936E-10	Heart- and neural crest derivatives-expressed protein 1
P57102	HAND2_DANRE	0.288	0.800211339	0.018	0.98648372	Heart- and neural crest derivatives-expressed protein 2

Supplementary Table 6. Significantly upregulated genes invovled in cell cycle, cell division and cell proliferations, related to Figure 2.

Protein ID	Protein Name	*C.hamatus/G.acuticeps*		*C.hamatus/T.bernacchii*		Description	GO Term
		Log2(CH/GA)	*P*(CH/GA)	Log2(CH/TB)	*P*(CH/TB)		
P49918	CDN1C_HUMAN	1.523	0.092	2.188	0.004	Cyclin-dependent kinase inhibitor 1C	GO:0007049
Q90459	CCND1_DANRE	2.355	NA	2.717	NA	G1/S-specific cyclin-D1	GO:0007049, GO:0051301
Q28270	CD34_CANFA	3.632	0.011	4.368	0.001	Hematopoietic progenitor cell antigen CD34	GO:0008283
Q00731	VEGFA_MOUSE	2.158	0.049	4.203	0.000	Vascular endothelial growth factor A	GO:0008283
Q9UJX2	CDC23_HUMAN	4.560	0.000	1.534	0.008	Cell division cycle protein 23 homolog	GO:0007049, GO:0051301
P51957	NEK4_HUMAN	2.724	0.001	3.945	0.000	Serine/threonine-protein kinase Nek4	GO:0007049, GO:0051301
Q3YBR2	TBRG1_HUMAN	5.166	0.000	4.929	0.000	Transforming growth factor beta regulator 1	GO:0007049
Q9UKG1	DP13A_HUMAN	2.293	0.000	7.036	0.000	DCC-interacting protein 13-alpha	GO:0008283
B1H1X4	SRRTA_XENLA	4.090	0.000	3.946	0.000	Serrate RNA effector molecule homolog A	GO:0008283
F1QFS9	UBP13_DANRE	7.143	0.000	4.543	0.000	Ubiquitin carboxyl-terminal hydrolase 13	GO:0008283
B5FYQ0	ARL3_TAEGU	2.583	0.035	5.495	0.000	ADP-ribosylation factor-like protein 3	GO:0007049, GO:0051301
Q9DAZ9	ZFY19_MOUSE	4.353	0.000	5.489	0.000	Zinc finger FYVE domain-containing protein 19	GO:0007049, GO:0051301
Q6DIS8	LZTS2_XENTR	4.454	0.000	3.654	0.000	Leucine zipper putative tumor suppressor 2 homolog	GO:0007049, GO:0051301
Q96RF0	SNX18_HUMAN	3.806	0.004	3.330	0.008	Sorting nexin-18	GO:0007049, GO:0051301
Q93008	USP9X_HUMAN	4.728	0.000	3.077	0.000	Probable ubiquitin carboxyl-terminal hydrolase FAF-X	GO:0007049, GO:0051301
Q7L8A9	VASH1_HUMAN	5.061	0.000	4.358	0.000	Vasohibin-1	GO:0007049
P19474	RO52_HUMAN	4.233	0.000	7.302	0.000	E3 ubiquitin-protein ligase TRIM21	GO:0007049
Q5E9N9	APEX2_BOVIN	3.183	0.000	4.319	0.000	DNA-(apurinic or apyrimidinic site) lyase 2	GO:0007049
Q9WVH3	FOXO4_MOUSE	2.836	0.069	2.409	0.106	Forkhead box protein O4	GO:0007049
O43903	GAS2_HUMAN	1.417	NA	3.928	NA	Growth arrest-specific protein 2	GO:0007049
P26696	MK01_XENLA	6.875	NA	6.004	NA	Mitogen-activated protein kinase 1	GO:0007049
Q283Q6	DBF4B_XENLA	3.009	0.025	2.796	0.027	Protein DBF4 homolog B	GO:0007049
Q5ZKG2	BRD7_CHICK	8.967	NA	8.593	NA	Bromodomain-containing protein 7	GO:0007049
Q8R0X2	CJ046_MOUSE	4.785	0.000	2.998	0.000	Uncharacterized protein C10orf46 homolog	GO:0007049
Q9Y219	JAG2_HUMAN	1.230	NA	1.880	NA	Protein jagged-2	GO:0007049
Q6DHG8	SKA1_DANRE	2.118	0.005	4.334	0.000	Spindle and kinetochore-associated protein 1	GO:0007049, GO:0051301
Q7Z5K2	WAPL_HUMAN	3.089	0.021	3.296	0.006	Wings apart-like protein homolog	GO:0007049, GO:0051301
Q3TIX9	SNUT2_MOUSE	2.778	0.000	2.866	0.000	U4/U6.U5 tri-snRNP-associated protein 2	GO:0007049, GO:0051301
Q7SXR3	MAEA_DANRE	3.269	0.000	2.421	0.000	Macrophage erythroblast attacher	GO:0007049, GO:0051301
Q99KW3	TARA_MOUSE	1.932	0.035	1.987	0.015	TRIO and F-actin-binding protein	GO:0007049, GO:0051301
B5X2S3	ARP8_SALSA	2.873	0.000	1.956	0.000	Actin-related protein 8	GO:0007049, GO:0051301

Q6NYW6	CLAP2_DANRE	2.137	0.005	1.527	0.033	CLIP-associating protein 2	GO:0007049, GO:0051301
O14578	CTRO_HUMAN	8.100	NA	7.627	NA	Citron Rho-interacting kinase	GO:0007049, GO:0051301
Q9ES70	NEK6_MOUSE	2.733	0.109	2.652	0.098	Serine/threonine-protein kinase Nek6	GO:0007049, GO:0051301
P42207	SEPT1_DROME	1.650	NA	1.163	NA	Septin-1	GO:0007049, GO:0051301
Q0P5A1	DCTN3_BOVIN	1.943	0.017	1.056	0.198	Dynactin subunit 3	GO:0007049, GO:0051301
Q7TME2	SPAG5_MOUSE	1.238	NA	1.019	NA	Sperm-associated antigen 5	GO:0007049, GO:0051301
Q9JK84	PAR6G_MOUSE	1.509	0.138	2.001	0.016	Partitioning defective 6 homolog gamma	GO:0007049, GO:0051301
P08754	GNAI3_HUMAN	1.363	0.168	1.588	0.055	Guanine nucleotide-binding protein G(k) subunit alpha	GO:0007049, GO:0051301
O75154	RFIP3_HUMAN	1.000	0.173	1.481	0.012	Rab11 family-interacting protein 3	GO:0007049, GO:0051301
P18754	RCC1_HUMAN	1.139	0.065	1.431	0.006	Regulator of chromosome condensation	GO:0007049, GO:0051301
Q28GU6	ARP19_XENTR	6.889	0.000	1.349	0.143	cAMP-regulated phosphoprotein 19	GO:0007049, GO:0051301
P38400	GNAI2_CANFA	2.882	0.005	2.724	0.004	Guanine nucleotide-binding protein G(i) subunit alpha-2	GO:0007049,GO:0051301, GO:0008283
O75410	TACC1_HUMAN	1.164	0.027	1.102	0.020	Transforming acidic coiled-coil-containing protein 1	GO:0007049,GO:0051301, GO:0008283
Q9Y5K6	CD2AP_HUMAN	5.957	NA	5.921	NA	CD2-associated protein	GO:0051301
Q90XC2	NEK8_DANRE	4.401	NA	5.130	NA	Serine/threonine-protein kinase Nek8	GO:0051301
Q86YI8	PHF13_HUMAN	5.281	NA	3.822	NA	PHD finger protein 13	GO:0051301
Q9UNH5	CC14A_HUMAN	4.515	NA	4.604	NA	Dual specificity protein phosphatase CDC14A	GO:0051301, GO:0008283
Q15742	NAB2_HUMAN	2.798	0.011	3.073	0.004	NGFI-A-binding protein 2	GO:0008283
Q9Z0M5	LICH_MOUSE	5.566	0.000	2.387	0.013	Lysosomal acid lipase/cholesteryl ester hydrolase	GO:0008283
Q5R7U4	GBG2_PONAB	6.914	0.000	2.117	0.029	Guanine nucleotide-binding protein G(I)/G(S)/G(O) subur	GO:0008283
Q16658	FSCN1_HUMAN	3.035	0.002	2.019	0.030	Fascin	GO:0008283

(GO:0007049 cell cycle;GO:0051301: cell division; GO:0008283: Cell proliferation)

Supplementary Table 7. qRT-PCR results for *bmp*2 and four selected genes involved in cell cycle process between the *C. hamatus* and *T. bernacchii*, related to Figure 7.

Gene name	qPCR analysis		
	Relative expression		$2^{-\triangle\triangle CT(CH/TB)}$
	△CT (CH/β-actin)	△CT (TB/β-actin)	
*bmp*2	4.562±0.631	10.543±0.198	63.130±0.667
bmp4	5.400±0.887	8.977±0.538	11.935±0.812
ccna2	2.889±0.327	6.286±0.429	10.533±0.718
ccnd1	2.356±0.399	4.389±0.447	32.686±0.624
ccnd2	4.567±0.592	5.432±0.380	1.821±0.233
cdc27	4.124±0.312	4.256±0.517	1.096±0.642

Note: The gene expression levels were shown by fold changes. *β-actin* was used as an internal control for qPCR analysis. All of the reactions were performed with three biological replicates, and each sample was assayed three times. The statistical significance was determined using a two-tailed unpaired Student *t*-test with $P < 0.05$.

Supplementary Table 9. The calculated expression levels of 6 selected miRNAs deduced from small RNA sequencing or quantitative real-time RT-PCR analysis, showing the consistency of differential expression of these miRNAs between *C. hamatus* and *T. bernacchii* detected by either method, related to Figure 7.

miRNA name	qPCR analysis			miRNA sequencing
	Relative expression		$2^{-\triangle\triangle CT(CH/TB)}$	log_2(CH/TB)
	△CT (CH/U6RNA)	△CT (TB/U6RNA)		
miR-144-5p	9.735±0.562	8.693±0.761	0.485±0.246	-4.941
miR-2188-5p	2.676±0.585	1.513±0.631	0.447±0.274	-5.398
miR-10c-5p	7.593±0.484	7.339±0.607	0.839±0.060	-1.184
miR-204-5p	9.345±0.304	8.551±0.211	0.577±0.187	-1.930
miR-458-3p	6.140±0.335	5.693±0.623	0.734±0.105	-2.366
miR-133-3p	0.913±0.688	2.200±0.274	2.440±0.303	1.390

Note: The gene expression levels were shown by fold changes. U6RNA was used as an internal control for qPCR analysis. All of the reactions were performed with three biological replicates, and each sample was assayed three times. The statistical significance was determined using a two-tailed unpaired Student *t*-test with $P < 0.05$.

Supplementary Table 10 .The list of the down -regulated miRNAs detected in the heart of the Antarctic icefish that target each of the 8 cardiac development marker genes, related to Table 1.

1. *bmp2*

miRNA Name	miRNA Sequence	Counts Per Million			*C.hamatus/G.acuticeps*	*C.hamatus/T.bernacchii*	Gene Target Prediction Value							
		C.hamatus	*G.acuticeps*	*T.bernacchii*	Log2(CH/GA)	Log2(CH/TB)	Tot Score	Tot Energy	Max Score	Max Energy	Strand	Len1	Len2	Positions
miR-2188-3p	GCTGTGTGAGGTCAGACCTATC	2	894	47	-7.877	-4.654	140	-14.87	140	-14.87	10	22	1430	508
miR-144-5p	AGGATATCATCTTATACTGTAAGT	1	367	138	-7.467	-7.089	113	-20.45	113	-20.45	8	24	1430	520
miR-204-5p	TTCCCTTTGTCATCCTATGCCT	14	122	67	-2.086	-2.269	132	-13.68	132	-13.68	73	22	1430	178
miR-458-3p	ATAGCTCTTTAAATGGTACTG	28	39	607	-1.047	-3.044	265	-28.66	134	-16.66	136	21	1430	391 1290

2.*mef2a*

miRNA Name	miRNA Sequence	Counts Per Million			*C.hamatus/G.acuticeps*	*C.hamatus/T.bernacchii*	Gene Target Prediction Value							
		C.hamatus	*G.acuticeps*	*T.bernacchii*	Log2(CH/GA)	Log2(CH/TB)	Tot Score	Tot Energy	Max Score	Max Energy	Strand	Len1	Len2	Positions
miR-144-5p	AGGATATCATCTTATACTGTAAGT	1	367	138	-7.467	-7.089	119	-15.28	119	-15.28	9	24	550	1
miR-204-5p	TTCCCTTTGTCATCCTATGCCT	14	122	67	-2.086	-2.269	135	-11.17	135	-11.17	74	22	550	173

3. *mef2c*

miRNA Name	miRNA Sequence	Counts Per Million			*C.hamatus/G.acuticeps*	*C.hamatus/T.bernacchii*	Gene Target Prediction Value							
		C.hamatus	*G.acuticeps*	*T.bernacchii*	Log2(CH/GA)	Log2(CH/TB)	Tot Score	Tot Energy	Max Score	Max Energy	Strand	Len1	Len2	Positions
miR-144-3p	TACAGTATAGATGATGTACTAT	9	297	346	-3.990	-5.247	264	-26.85	134	-14.61	3	22	3216	348 2707
miR-144-5p	AGGATATCATCTTATACTGTAAGT	1	367	138	-7.467	-7.089	131	-14.69	131	-14.69	30	24	3216	667
miR-2188-5p	AAGGTCCAACCTCACATGTCCT	14	2231	807	-6.334	-5.901	138	-22.09	138	-22.09	48	22	3216	3138
miR-204-5p	TTCCCTTTGTCATCCTATGCCT	14	122	67	-2.086	-2.269	414	-50.31	152	-18.82	75	22	3216	155 2915 713
miR-182-5p	TTTGGCAATGGTAGAACTCACA	1	17	16	-3.000	-3.939	675	-72.72	137	-16.75	93	22	3216	642 740 1476 1101 2715

4. *gata4*

miRNA Name	miRNA Sequence	Counts Per Millionr			*C.hamatus/G.acuticeps*	*C.hamatus/T.bernacchii*	Gene Target Prediction Value							
		C.hamatus	*G.acuticeps*	*T.bernacchii*	Log2(CH/GA)	Log2(CH/TB)	Tot Score	Tot Energy	Max Score	Max Energy	Strand	Len1	Len2	Positions
miR-144-3p	TACAGTATAGATGATGTACTAT	9	297	346	-3.990	-5.247	442	-39.5	158	-13.62	4	22	1480	314 162 1320
miR-2188-3p	GCTGTGTGAGGTCAGACCTATC	2	894	47	-7.877	-4.654	132	-18.14	132	-18.14	13	22	1480	960
miR-144-5p	AGGATATCATCTTATACTGTAAGT	1	367	138	-7.467	-7.089	276	-22.14	144	-11.83	31	24	1480	755 1234
miR-204-5p	TTCCCTTTGTCATCCTATGCCT	14	122	67	-2.086	-2.269	131	-17.17	131	-17.17	76	22	1480	478
miR-182-5p	TTTGGCAATGGTAGAACTCACA	1	17	16	-3.000	-3.939	143	-8.84	143	-8.84	94	22	1480	421

5. *gata6*

miRNA Name	miRNA Sequence	Counts Per Million			*C.hamatus/G.acuticeps*	*C.hamatus/T.bernacchii*	Gene Target Prediction Value							
		C.hamatus	*G.acuticeps*	*T.bernacchii*	Log2(CH/GA)	Log2(CH/TB)	Tot Score	Tot Energy	Max Score	Max Energy	Strand	Len1	Len2	Positions
miR-144-3p	TACAGTATAGATGATGTACTAT	9	297	346	-3.990	-5.247	139	-13.32	139	-13.32	5	22	771	351
miR-144-5p	AGGATATCATCTTATACTGTAAGT	1	367	138	-7.467	-7.089	131	-7.88	131	-7.88	32	24	771	567
miR-2188-5p	AAGGTCCAACCTCACATGTCCT	14	2231	807	-6.334	-5.901	145	-18.01	145	-18.01	113	21	771	269

6. *tbx2*

miRNA Name	miRNA Sequence	Counts Per Million			*C.hamatus/G.acuticeps*	*C.hamatus/T.bernacchii*	Gene Target Prediction Value							
		C.hamatus	*G.acuticeps*	*T.bernacchii*	Log2(CH/GA)	Log2(CH/TB)	Tot Score	Tot Energy	Max Score	Max Energy	Strand	Len1	Len2	Positions
miR-144-3p	TACAGTATAGATGATGTACTAT	9	297	346	-3.990	-5.247	282	-23.82	145	-12.76	6	22	1329	973 332
miR-2188-3p	GCTGTGTGAGGTCAGACCTATC	2	894	47	-7.877	-4.654	275	-36.43	145	-21.01	15	22	1329	550 559
miR-144-5p	AGGATATCATCTTATACTGTAAGT	1	367	138	-7.467	-7.089	143	-11.35	143	-11.35	33	24	1329	352

7.*tbx20*

miRNA Name	miRNA Sequence	Counts Per Million			*C.hamatus/G.acuticeps*	*C.hamatus/T.bernacchii*	Gene Target Prediction Value							
		C.hamatus	*G.acuticeps*	*T.bernacchii*	Log2(CH/GA)	Log2(CH/TB)	Tot Score	Tot Energy	Max Score	Max Energy	Strand	Len1	Len2	Positions
miR-10c-5p	TACCCTGTAGATCCGGATTTGT	72	659	204	-2.186	-1.526	284	-35.12	143	-19.21	70	22	780	701 321
miR-182-5p	TTTGGCAATGGTAGAACTCACA	1	17	16	-3.000	-3.939	134	-12.07	134	-12.07	97	22	780	597
miR-204-5p	TTCCCTTTGTCATCCTATGCCT	14	122	67	-2.086	-2.269	278	-39.55	146	-20.39	79	22	780	344 121
miR-2188-5p	AAGGTCCAACCTCACATGTCCT	14	2231	807	-6.334	-5.901	131	-26.16	131	-26.16	52	22	780	248

8. *hand2*

miRNA Name	miRNA Sequence	Counts Per Million			*C.hamatus/G.acuticeps*	*C.hamatus/T.bernacchii*	Gene Target Prediction Value							
		C.hamatus	*G.acuticeps*	*T.bernacchii*	Log2(CH/GA)	Log2(CH/TB)	Tot Score	Tot Energy	Max Score	Max Energy	Strand	Len1	Len2	Positions
miR-144-3p	TACAGTATAGATGATGTACTAT	9	297	346	-3.990	-5.247	131	-9.79	131	-9.79	9	22	604	260
miR-2188-3p	GCTGTGTGAGGTCAGACCTATC	2	894	47	-7.877	-4.654	288	-41.5	158	-23.47	18	22	604	429 439

Supplementary Table 11. MiRNA-458-3p and miR-144-5p antagomir microinjection, heart size measurement and the relevant statistical analysis results, related to Figure 4.

Atrium（μ m2）									
	WT	NC	miR-458-3p	miR- 144-5p	NC/WT *P* value	miR-458-3p/WT*P* value	miR-458-3p/NC *P* value	miR- 144-5p/WT *P* value	miR- 144-5p/NC *P* value
1	27106	19450	29407	31440					
2	25703	22025	31485	27859					
3	26879	27989	32189	25557					
4	22728	29306	26913	31337					
5	26653	30498	29923	31382					
6	30170	36798	31370	29628					
7	27692	20342	34300	25334					
8	25825	23457	26827	27311					
9	29933	23608	30287	26106	*P* =0.925844	*P* =0.00038**	*P* =0.009538**	*P* =0.068432	*P* =0.302799
10	25746	24066	34913	28774					
11	26197	27988	30205	24049					
12	29378	27049	31435	32264					
13	24885	29453	31249	26850					
14	26566	32228	29584	31507					
15	28974	32099	35525	29564					
Average± SD	26962±2018	27090±4881	31041±2530	28597±2664					

Ventricle（μ m2）									
	WT	NC	miR-458-3p	miR- 144-5p	NC/ WT *P* value	miR-458-3p/WT *P* value	miR-458-3p/NC *P* value	miR- 144-5p/WT *P* value	miR- 144-5p/NC *P* value
1	32114	28564	32234	33321					
2	22105	23615	35830	40229					
3	30813	32363	35505	27496					
4	23751	30745	31801	29445					
5	26280	40932	31730	36926					
6	35242	38224	41996	38494					
7	34571	34243	45668	36846					
8	31481	23478	26630	30670					
9	23883	28332	42517	38513	*P* =0.088483	*P* =0.000297**	*P* =0.036305*	*P* =0.000305**	*P* =0.022725*
10	27202	25411	40587	53958					
11	24155	29130	38407	32419					
12	29613	28607	27297	40034					
13	19578	41310	32248	32824					
14	25305	32497	39401	32618					
15	31425	30087	33249	45995					
Average± SD	27835±4727	31169±5586	35673±5633	36653±6811					

The statistical significance was determined using a two-tailed unpaired Student’s t-test with P < 0.05.

Supplementary Table 12. qRT-PCR results for miR-458-3p (miR-144-5p) between the embryo fish heart micro-injected by miR-458-3p antagomir (miR-144-5p antagomir), the fish heart micro-injected with NC antagomir and the WT fish heart, related to Figure 4.

miRNA name	qPCR analysis			
	Relative expression		$2^{-\triangle\triangle CT(miRNA/WT)}$	*P* Value
	△CT (miRNA/U6RNA)	△CT (WT/U6RNA)		
miR-458-3p	9.634±0.548	7.157±0.499	0.190±0.030	*P* value (miR-458/WT)=0.001**
NC (miR-458-3p)	7.812±0.272	7.157±0.499	0.667±0.130	*P* value (miR-458/NC)=0.034*
				P value (NC/WT)=0.102
miR-144-5p	15.582±0.201	12.438±0.653	0.156±0.061	*P* value (miR-144-5p/WT)=0.001**
NC(miR-144-5p)	13.371±0.327	12.438±0.653	0.549±0.165	*P* value (miR-144-5p/NC)=0.047*
				P value (NC/WT)=0.112

Note: Small RNAs from total RNA preparations of miR-458-3p antagomir micro-injected, NC antagomir micro-injected, and WT zebrafish embryos heart tissues were isolated. The gene expression level was shown by fold changes. U6RNA was used as an internal control for qPCR analysis. The reactions were performed with three biological replicates, and each sample was assayed three times. The statistical significance was determined using a two-tailed unpaired Student *t*-test with $^*P < 0.05$ ($^{**}P<0.01$).

Supplementary Table 14. qRT-PCR results for miR-458-3p, miR-144-5p and *bmp2* between the hypoxia acclimated zebrafish heart and the normoxia cultivated zebrafish heart, related to Figure 7.

miRNA name	qPCR analysis		
	Relative expression		$2^{-\triangle\triangle CT(Hypoxia/Normoxia)}$
	△CT (Hypoxia/U6)	△CT (Normoxia/U6)	
Dre-miR-458-3p	5.176±0.307	3.281±0.140	0.269±0.031
Dre-miR-144-5p	7.533±0.939	5.333±0.822	0.251±0.025
	△CT (Hypoxia/β-actin)	△CT (Normoxia/β-actin)	
bmp2	9.400±0.698	11.032±0.697	3.102±0.105

Note: Small RNAs from total RNA preparations of hypoxia acclimated or normoxia zebrafish heart tissues were isolated. The gene expression level was shown by fold changes. U6RNA was used as an internal control for qPCR analysis. The reactions were performed with three biological replicates, and each sample was assayed three times. The statistical significance was determined using a two-tailed unpaired Student *t*-test with $P < 0.05$.

Supplementary Table 15. qRT-PCR results for seven selected genes involved in cell cycle process between the hypoxia acclimated zebrafish heart and the normoxia cultivated zebrafish heartrelated to Figure 7.

Gene name	qPCR analysis		
	Relative expression		$2^{-\triangle\triangle CT(Hypoxia/Normoxia)}$
	△CT (Hypoxia/β-actin)	△CT (Normoxia/β-actin)	
ccna2	4.243±0.408	9.100±0.519	29.094±2.719
ccnb1	5.686±0.727	8.300±0.779	6.126±0.241
ccnd1	5.398±0.847	6.667±1.034	2.440±0.406
ccnd2	3.063±0.710	4.500±0.787	2.728±0.284
cdc2	8.928±0.805	10.530±0.912	3.045±0.240
cdc20	5.215±0.924	6.600±1.023	2.647±0.437
cdc27	6.764±1.047	8.571±0.836	3.537±0.538

Note: The gene expression levels were shown by fold changes. *β-actin* was used as an internal control for qPCR analysis. All of the reactions were performed with three biological replicates, and each sample was assayed three times. The statistical significance was determined using a two-tailed unpaired Student *t*-test with $P < 0.05$.

Supplementary Table 16. qRT-PCR results for miR-458-3p, miR-144-5p and *bmp2* between the hypoxia stressed *T. bernacchii* heart and the normoxia *T. bernacchii* heart, related to Figure 7.

miRNA name	qPCR analysis		
	Relative expression		$2^{-\triangle\triangle CT(Hypoxia/Normoxia)}$
	△CT (Hypoxia/U6)	△CT (Normoxia/U6)	
miR-458-3p	7.732±0.655	6.206±0.466	0.350±0.046
miR-144-5p	5.700±0.455	4.105±0.484	0.331±0.017
	△CT (Hypoxia/β-actin)	△CT (Normoxia/β-actin)	
bmp2	9.367±0.419	9.747±0.387	1.302±0.045

Note: Small RNAs from total RNA preparations of hypoxia acclimated or normoxia *T. bernacchii* heart tissues were isolated. The gene expression level was shown by fold changes. U6RNA was used as an internal control for qPCR analysis. The reactions were performed with three biological replicates, and each sample was assayed three times. The statistical significance was determined using a two-tailed unpaired Student *t*-test with $P < 0.05$.

Supplementary Table 17. Summary of qPCR primers, related to Figure 7.

Gene	Forward	Reverse	Size
Chionodraco hamatus			
bmp2	5' GTAGGCTGGCACAAGTGGAT 3'	5' CACGGCTCCATTCACAGAGT 3'	147bp
bmp4	5'GAACTCTACCAACCACGCCA 3'	5' GCAGCCCTCTACCACCATTT 3'	169bp
cyclina2 (*ccna2*)	5' AGCCTACACTCTGGCAAACC 3'	5' TCGAACAGACTGCTGTGCAT 3'	157bp
cyclin d2 (*ccnd2*)	5' CTTCATCGCCCTATGTGCCA 3'	5' CTGCTCCTGGCACTCTTTCA 3'	199bp
cell division cycle protein 27 (*cdc27*)	5' TAAATGCTGCGTGGAGCTGA 3'	5' TGTGTCCCAGTAACGACAGC 3'	143bp
β-actin	5' ACGTGGCTCTGGACTTTGAG 3'	5' CCGATGGTGATGACCTGTCC 3'	100bp
Trematomus bernacchii			
bmp2	5' ATGTGGGTTGGAACGAGTGG 3'	5' GACTGAGTTGACCAGCGTCT 3'	137bp
bmp4	5'GAACTCTACCAACCACGCCA 3'	5' GCAGCCCTCTACCACCATTT 3'	169bp
cyclina2 (*ccna2*)	5' AGCCTACACTCTGGCAAACC 3'	5' TCGAACAGACTGCTGTGCAT 3'	157bp
cyclin d2 (*ccnd2*))	5' TGGAATCGGACATAGCCGTG 3'	5' ACACCTCGTGCATCCAAACT 3'	183bp
cell division cycle protein 27 (*cdc27*)	5' TCGTACCGAGTGGAAGGGAT 3'	5' ACTTGATGGCGATGTCGTGT 3'	178bp
β-actin	5' GGAAGATGAAATCGCCGCAC 3'	5' AACGTAGCTGTCCTTCTGGC 3'	160bp
Danio rerio			
bmp2	5' TGTACGTGGACTTCAGCGAC 3'	5' ACCAGTGTCTGCACCATAGC 3'	145bp
cyclin d1 (*ccnd1*)	5' TGCGACAGACGTCAACTTCA 3'	5' ATGAGAGGCAACTGTCGGTG 3'	110bp
cyclina2 (*ccna2*)	5' CGCGGTTCATGACTCCGATA 3'	5' CTCTGGCTGTTCTCCAGCTC 3'	159bp
cyclin d2 (*ccnd2*))	5' AGAACTGCTGGAATGGGAGC 3'	5' GCTCCAGCTTATCCTCAGGC 3'	125bp
g2/mitotic-specific cyclin-b1(*ccnb1*)	5' GCAGTTGTAGCGAACAAGCC 3'	5' GAACCACAGGTGCCTTCTCA 3'	148bp
cell division control protein 2(*cdc2*)	5'TCGCCACCAAGAAACCTCTC 3'	5' AAGGTCGATGCCGTTCTTGT 3'	200bp
cell division cycle protein 20 (*cdc20*)	5' AAGAATTGGTGTCGGGCCAT 3'	5' GGAACCGTCTGGACTCAGTG 3'	134bp
cell division cycle protein 27 (*cdc27*)	5' AGGCTCTATGCTGAGGTCCA 3'	5' TCCTCCGGTCAGGATCTGTT 3'	198bp
β-actin	5' ACGGCCAAGTCATCACCATT 3'	5' TGATGCTGTTGTAGGCGGTT 3'	117bp

Supplementary Table 18. Summary of miRNA qPCR primers, related to Figure 7.

microRNA	Primer Sequences
To amplify miRNAs from C. hamatus and T. bernacchii	
U6 RNA	5’ AGGATGACACGCAAATCCGTG 3’
miR-144-5p	5’ AGGATATCATCTTATACTGTAAGT 3’
miR-2188-5p	5’ AAGGTCCAACCTCACATGTCCT 3’
miR-10c-5p	5’ TACCCTGTAGATCCGGATTTGT 3’
miR-204-5p	5’ TTCCCTTTGTCATCCTATGCCT 3’
miR-458-3p	5’ ATAGCTCTTTAAATGGTACTG 3’
miR-133a-3p	5’ TTGGTCCCCTTCAACCAGCTGT 3’
Universal Reverse	5’ ATTCTAGAGGCCGAGGCGG 3’
To amplify miRNAs from Danio rerio	
Dre-U6 RNA	5’ AGGATGACACGCAAATCCGTG 3’
Dre-miR-458-3p	5’ ATAGCTCTTTGAATGGTACTG 3’
Dre-miR-144-5p	5’ AGGATATCATCTTATACTGTAAGT 3’
Universal Reverse	5’ ATTCTAGAGGCCGAGGCGG 3’
